# The SIRT3-ATAD3A axis regulates MAM dynamics and mitochondrial calcium homeostasis in cardiac hypertrophy

**DOI:** 10.7150/ijbs.89253

**Published:** 2024-01-01

**Authors:** Zeyu Li, Ou Hu, Suowen Xu, Chenjia Lin, Wenjing Yu, Dinghu Ma, Jing Lu, Peiqing Liu

**Affiliations:** 1 National and Local United Engineering Lab of Druggability and New Drugs Evaluation, Guangdong Provincial Key Laboratory of New Drug Design and Evaluation, School of Pharmaceutical Sciences, Sun Yat-sen University, Guangzhou 510006, China.; 2 Guangdong Province Engineering Laboratory for Druggability and New Drug Evaluation, School of Pharmaceutical Sciences, Sun Yat-sen University, Guangzhou 510006, China.; 3 Department of Endocrinology, Institute of Endocrine and Metabolic Diseases, The First Affiliated Hospital of USTC, Division of Life Sciences and Medicine, Clinical Research Hospital of Chinese Academy of Sciences (Hefei), University of Science and Technology of China, Hefei, 230001, China.

**Keywords:** Mitochondria-associated membranes, Mitochondria, IP3R1-GRP75-VDAC1 complex, Cardiac hypertrophy, ER stress, acetylation

## Abstract

Mitochondria are energy-producing organelles that are mobile and harbor dynamic network structures. Although mitochondria and endoplasmic reticulum (ER) play distinct cellular roles, they are physically connected to maintain functional homeostasis. Abnormal changes in this interaction have been linked to pathological states, including cardiac hypertrophy. However, the exact regulatory molecules and mechanisms are yet to be elucidated. Here, we report that ATPase family AAA-domain containing protein 3A (ATAD3A) is an essential regulator of ER-mitochondria interplay within the mitochondria-associated membrane (MAM). ATAD3A prevents isoproterenol (ISO)-induced mitochondrial calcium accumulation, improving mitochondrial dysfunction and ER stress, which preserves cardiac function and attenuates cardiac hypertrophy. We also find that ATAD3A is a new substrate of NAD^+^-dependent deacetylase Sirtuin 3 (SIRT3). Notably, the heart mitochondria of SIRT3 knockout mice exhibited excessive formation of MAMs. Mechanistically, ATAD3A specifically undergoes acetylation, which reduces self-oligomerization and promotes cardiac hypertrophy. ATAD3A oligomerization is disrupted by acetylation at K134 site, and ATAD3A monomer closely interacts with the IP3R1-GRP75-VDAC1 complex, which leads to mitochondrial calcium overload and dysfunction. In summary, ATAD3A localizes to the MAMs, where it protects the homeostasis of ER-mitochondria contacts, quenching mitochondrial calcium overload and keeping mitochondrial bioenergetics unresponsive to ER stress. The SIRT3-ATAD3A axis represents a potential therapeutic target for cardiac hypertrophy.

## Introduction

The number of patients with cardiac hypertrophy is rising due to population aging, affecting millions worldwide. Cardiac hypertrophy is a complex and debilitating clinical syndrome with an estimated monetary cost to the healthcare system 1, 2. Despite significant progress in recent decades, the pathophysiology of cardiac hypertrophy has yet to be fully understood. Endoplasmic reticulum (ER) and mitochondrial dysfunction have emerged as critical events in the alterations that occur after hypertrophic stress 3, 4. Unbalance between Excitation-contraction coupling and the mitochondrial energy supply are potential contributors to cardiac hypertrophy progression 5, 6. In addition, the uptake of cytosolic calcium into the mitochondrial matrix controls crucial Krebs cycle and electron transport chain enzymes and is required for synchronizing the heart's energy supply 7. However, calcium is mainly stored in the ER as a highly dynamic and intracellular signal 8. Through the functional interaction of calcium transport channels, calcium buffers, and calcium transport organelles such as the ER, intracellular calcium homeostasis is maintained 9, 10. ER stress develops as a consequence of disturbances in ER calcium homeostasis, such as ER calcium depletion. It has been established that mitochondria, as the primary energy source of ATP, are closely interconnected with the ER 11. Calcium leakage from the ER could cause oxidative stress and altered membrane potential in mitochondria, which causes mitochondrial depolarization and excessive fission 12. Therefore, disruption of ER-to-mitochondria (ER-mito) communication homeostasis may have important pathological implications in the development of cardiac hypertrophy. However, the underlying mechanism is still largely unknown.

The metabolic effects of ER and mitochondrial malfunction have been evaluated and investigated independently 13-15. These organelles physically and functionally interact and can govern one another's function 16-18. Mitochondria-associated ER membrane (MAMs) are defined as contacts between the ER and mitochondria 19, which are reported to be part of autophagy, steroid synthesis and phospholipid metabolism. The importance of crosstalk between the ER and mitochondria has been reported mainly in neurodegenerative diseases 20. Our study focuses on MAM-associated cellular pathways for ER-mitochondrial calcium trafficking. To increase mitochondrial calcium levels, ER calcium is released through IP3 Receptor 1 (IP3R1) and transported to the outer mitochondrial membrane (OMM) via voltage-dependent anion channel 1 (VDAC1) 21. Calcium ultimately enters the inner mitochondrial membrane (IMM) via the mitochondrial calcium uniporter (MCU) 22. Calcium can activate mitochondrial ATPase, and calcium transfer from the endoplasmic reticulum to the mitochondria boosts calcium-sensitive tricarboxylic acid (TCA) cycle enzymes, which ultimately results in an increase in mitochondrial metabolism 23. It has also been reported that cytochrome C exits the mitochondria and binds to IP3R1 at MAMs, amplifying calcium-dependent apoptosis 24. Given that MAM proteins are crucial for regulating mitochondrial respiration and cellular viability as well as maintaining calcium homeostasis between the ER and mitochondria, we speculate that changes in MAMs and its mediated calcium transport may be involved in the pathological process of myocardial hypertrophy 25, 26.

We and other researchers have reported that SIRT3, as the most important sirtuin in mitochondria, can play an anti-pathological role in cardiac hypertrophy 27. It plays a crucial role in the regulation of mitochondrial function and antioxidant capacity 28. SIRT3 has some substrate proteins that are involved in MAM formation, as evidenced by pharmacological inhibition of cyclophilin D (CypD), leading to disruption of MAM integrity in mice 29, 30. Various studies have also confirmed that SIRT3 exerts regulatory effects on calcium homeostasis 31, 32. In 2015, Bochaton first reported that SIRT3 regulated calcium retention capacity via modification of the CypD acetylation status 33. More recently, Gao et al. reported the inhibitory effects of SIRT3 on mitochondrial calcium uniporter (MCU)-mediated mitochondrial calcium overload 34. Calcium uptake in mitochondria plays a pivotal role in controlling the formation of reactive oxygen species (ROS) in mitochondria. Excess mitochondrial calcium and ROS induce the mitochondrial permeability transition pore (mPTP) to open, resulting in cardiomyocyte apoptosis. Therefore, orchestrated transfer of calcium at MAMs is required for the maintenance of mitochondrial energy production and membrane stability. Although SIRT3 is the most important deacetylase involved in mitochondrial energy homeostasis, far less is known about the effect of SIRT3 on MAM-mediated mitochondrial calcium uptake.

The mitochondrial membrane-anchored protein ATPase family AAA-domain containing protein 3A (ATAD3A) is nuclear-encoded and involved in a variety of functions, including mitochondrial dynamics, mitochondrial DNA organization, and cholesterol metabolism 35,36. Previous studies have shown that ATAD3A suppresses hyperactivated mitochondrial fragmentation and plays a key role in mitochondrial ultrastructure integrity 37,38. Silencing ATAD3A lowers rates of basal oxygen consumption and decreases oxidative capability. As a result, the appropriate function of ATAD3A is crucial for mitochondrial morphology and energy supply. In addition, mutations in the ATAD3A gene also lead to altered mitochondrial-ER interactions and neurological syndromes in humans 39-41. Fifty amino acids at ATAD3A's N-terminus have been postulated to form an ER-insertion motif. In MA-10 cells, inhibition of ATAD3A activity decreased steroid hormone-induced progesterone production, suggesting a role of ATAD3A in MAM formation 42. Given its unique localization on MAMs, ATAD3A might regulate ER-to-mito calcium transfer, and its mechanism deserves further investigation.

To date, the vital role of ATAD3A in chronic heart remodeling has not been studied in detail. In the present work, ATAD3A acetylation increased and aberrant MAM production was induced by SIRT3 depletion during the hypertrophic process. We first identified SIRT3 as the major deacetylase of ATAD3A. We show that hypertrophic stimuli drive an abnormal increase in the binding affinity of the IP3R1-GRP75-VDAC1 complex, which results in increased IP3R1-mediated calcium flux from the ER to mitochondria in the heart. Increased mitochondrial ROS generation and disruption of metabolic balance accompany mitochondrial calcium excess. Cardio-specific ATAD3A overexpression protects against diastolic dysfunction and morphological changes in MAMs. ATAD3A-expressing cardiomyocytes exhibited decreased generation of IP3R1/GRP75/VDAC1 complexes and decreased IP3R1-stimulated calcium transport to mitochondria. Our work uncovers the potential of ATAD3A as a therapeutic target for cardiac hypertrophy.

## Results

### Increased levels of ATAD3A acetylation upon hypertrophic stimulation

**Figure 1:**
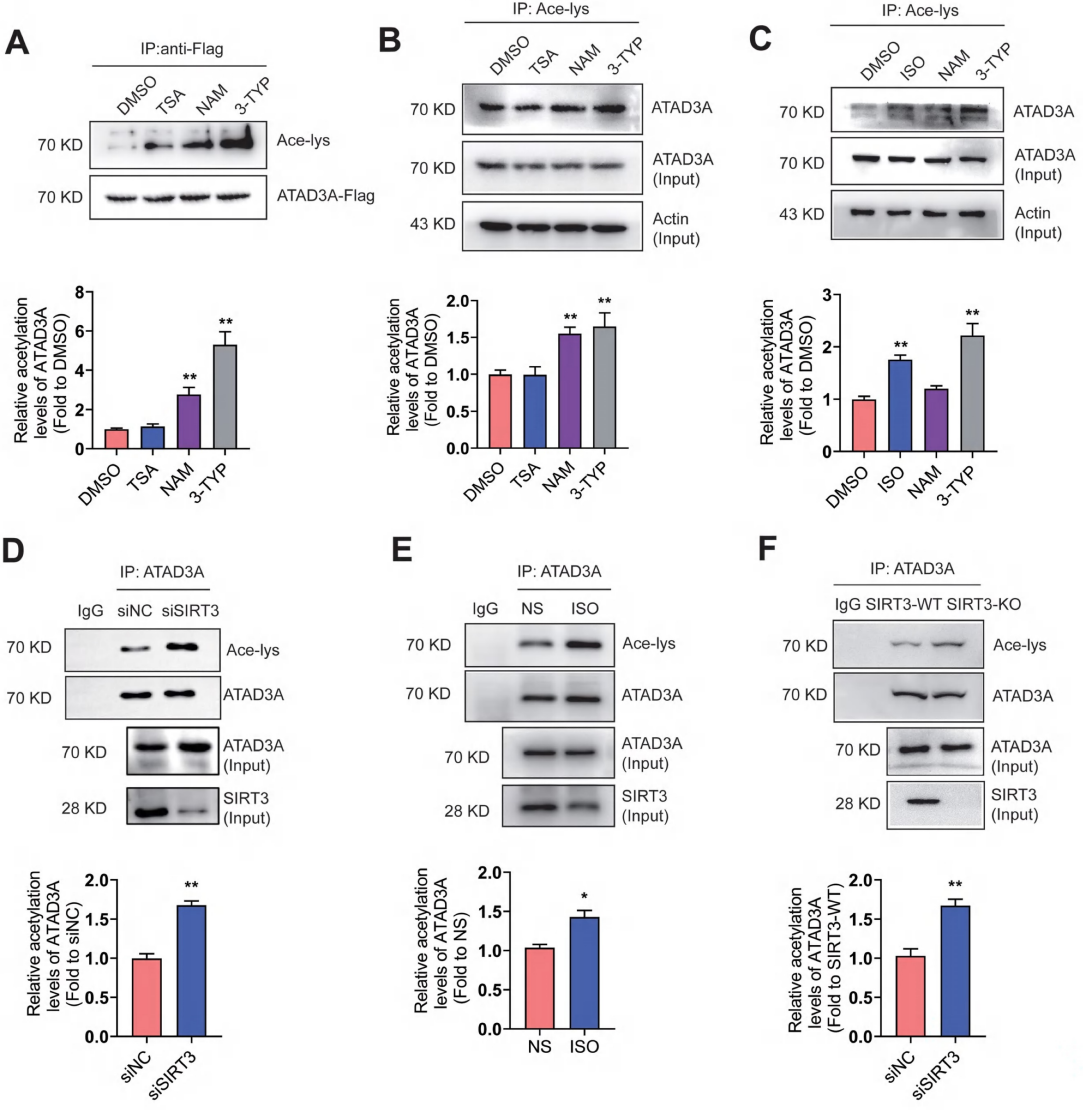
** ATAD3A acetylation increases in response to hypertrophic stimulation.** (A) Acetylation of exogenous ATAD3A in HEK293 cells in the presence of TSA (100 nM, 24 h), NAM (5 mM, 24 h) and 3-TYP (50 μM, 24 h). (B) The acetylation signal of endogenous ATAD3A in HEK293 cells was analyzed by immunoprecipitation. (C) Acetylation of ATAD3A in NRCMs in the presence of ISO (10 μM, 24 h), NAM (5 mM, 24 h) and 3-TYP (50 μM, 24 h). (D) Knockdown of SIRT3 by siRNA increases acetylation of ATAD3A. (E) Analysis of acetylation of ATAD3A in the cardiac tissue of Sprague Dawley (SD) rats injected with ISO (5 mg.kg^-1^.d^-1^) or normal saline (NS) for one week. (F) Analysis of acetylation of ATAD3A in cardiac tissue from the SIRT3-WT or SIRT3-KO groups. The data are presented as the mean ± SEM. ^**^P < 0.01, n = 3. Ace-lys, acetylated lysine; ISO, isoproterenol; TSA, trichostatin A; NAM, nicotinamide; NS, normal saline.

ATAD3A is related to multiple mitochondrial processes, including nucleoid organization and cholesterol metabolism. To test whether ATAD3A is an acetylated protein, the acetylation of exogenously expressed ATAD3A (Figure 1A) and endogenous ATAD3A in HEK293T cells (Figure 1B) were evaluated. HEK293T cells were treated with trichostatin A (TSA, HDAC inhibitor), nicotinamide (NAM, SIRT1 inhibitor), or 3-TYP (SIRT3 inhibitor). Strong acetylation of ATAD3A was detected in NAM-treated and 3-TYP-treated cells but not in TSA-treated cells. In neonatal rat cardiomyocytes (NRCMs), ISO, a classic stimulus for cardiac hypertrophy, stimulated acetylation of endogenous ATAD3A, similar to NAM and 3-TYP (Figure 1C). Among these SIRT family proteins, the expression of SIRT3 changes the most upon ISO stimulation (Figure S2). We previously reported that SIRT3 inhibits cardiac hypertrophy and ISO stimulation resulted in downregulation of SIRT3 expression 43. Knockdown of SIRT3 by RNA interference caused markedly elevated acetylation of ATAD3A (Figure 1D). The similar result was obtained in heart lysates from ISO-stimulated mice and SIRT3 knockout mice (Figure 1E and Figure 1F). However, we observed no significant change in ATAD3A protein or mRNA levels after stimulation of NRCMs with ISO over various time points (Figure S1). These findings indicate that ATAD3A may be an acetylated protein, and acetylation of ATAD3A may be involved in the progression of pathological cardiac hypertrophy.

### ATAD3A is deacetylated at the K134 site by SIRT3

**Figure 2:**
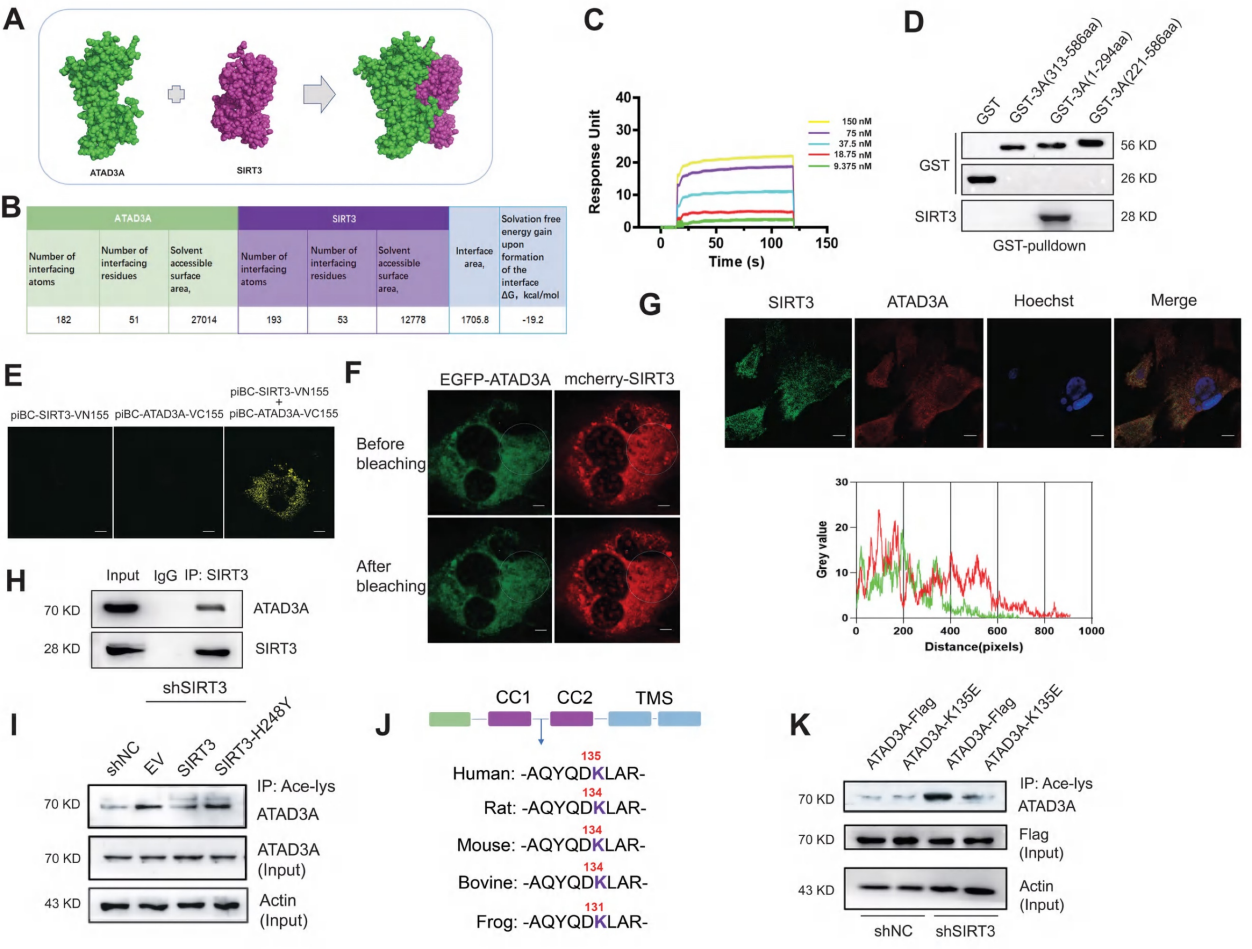
** Interaction of ATAD3A and the deacetyltransferase SIRT3.** (A) The SIRT3-ATAD3A docking result was visualized by PyMOL software. (B) Statistics of the interface between SIRT3 and ATAD3A according to PDBePISA. (C) Interactions between SIRT3 and ATAD3A were determined by SPR assay. (D) Identification of the interaction between SIRT3 and truncated ATAD3A domains by GST pull-down. GST alone and GST fusion proteins were affinity-purified before pulldown assay with rat heart lysate. (E) Interactions were visualized in NRCMs by bimolecular fluorescence complementation assay (BiFC). Representative images of four independent samples are shown (scale bar =5 μm). (F) NRCMs were transfected with EGFP-ATAD3A and mCherry-SIRT3 separately. The region of interest (as shown by the circled area) was irradiated with a 750 nm laser using two-photon excitation to achieve photobleaching. Representative images of four independent samples are shown (scale bar =5 μm). (G) Confocal images of SIRT3 (green) and ATAD3A (red) immunofluorescence colocalization (scale bar =10 μm). Gray value analysis of the SIRT3 and ATAD3A signals is shown. (H) Co-IP shows that SIRT3 interacts with ATAD3A in mouse heart tissue. (I) ATAD3A acetylation in cardiomyocytes transfected with Flag-tagged pcDNA3.1-SIRT3 or SIRT3-H248Y 48 h after SIRT3 shRNA infection. (J) Comparison of sequence homology analysis of ATAD3A in different species. K135 is well conserved across species. (K) ATAD3A acetylation in HEK293 cells transfected with WT-ATAD3A or ATAD3A-K135E 48 h after SIRT3 shRNA infection. FRET, fluorescence resonance energy transfer; BiFC, bimolecular fluorescence complementation assay; CO-IP, coimmunoprecipitation.

We tested whether ATAD3A is a substrate for SIRT3-mediated deacetylation. Homology models of ATAD3A were generated in SWISS-MODEL. The SIRT3-ATAD3A complex was computationally predicted using Z-DOCK 3.0.2 (Figure 2A), and the interface residues of the best SIRT3-ATAD3A complex were analyzed by the PDBePISA web server. The structure of the SIRT3-ATAD3A complex demonstrates the formation of an intimate interface between these proteins, which covers 19.5% of the solvent-accessible surface area of the effector (1705.8 Å^2^, Figure 2B). There were three salt bridges and seven hydrogen bonds in the interface. Then, we examined the kinetics of ATAD3A binding with SIRT3. Using surface plasmon resonance (SPR), multicycle kinetics were carried out. The equilibrium dissociation constant (Kd) was 50.9 nM, according to the data (Figure 2C). The truncation experiments identified that SIRT3 bound to the N-terminal region of ATAD3A (Figure 2D). Bimolecular fluorescence complementation (BiFC) assays in NRCMs showed the interaction between SIRT3 and ATAD3A (Figure 2E). Validation of interactions comes from fluorescence resonance energy transfer (FRET) measurements by acceptor photobleaching (Figure 2F). Confocal microscopy revealed the interaction between ATAD3A and SIRT3 in NRCMs (Figure 2G). Moreover, endogenous ATAD3A and SIRT3 co-IP in lysates from mouse hearts (Figure 2H) indicate that the SIRT3-ATAD3A complex occurs *in vivo*. The efficiency of shRNA‐mediated SIRT3 knockout was checked by Western blotting (Figure S2A). Knockdown of SIRT3 in cardiomyocytes primarily increased the basal acetylation level of ATAD3A. The increase could be reversed by reintroducing WT-SIRT3 but not acetyltransferase-deficient SIRT3 (SIRT3-H248Y) into SIRT3 knockdown cells (Figure 2I). Moreover, the overexpression and interference of SIRT3 did not affect ATAD3A expression (Figure S2B). The bioinformatics prediction website PAIL (Prediction of Acetylation on Internal Lysines) identified that ATAD3A could be acetylated at the K44, K135, K231, K442 and K549 sites. However, only K135 resides in the CC domain of the N-terminal region of ATAD3A and is also highly conserved among species (Figure 2J). To verify this, we used mutant K135E (replaced lysine with glutamic acid) to mimic deacetylated ATAD3A. WT-ATAD3A and ATAD3A-K135E plasmids were transfected into HEK293 cells. Compared with ATAD3A-Flag, ATAD3A-K135E showed markedly reduced acetylation (Figure 2K). These data suggest that SIRT3 may be a deacetylase of ATAD3A.

### SIRT3 deacetylates ATAD3A to regulate its oligomerization

**Figure 3:**
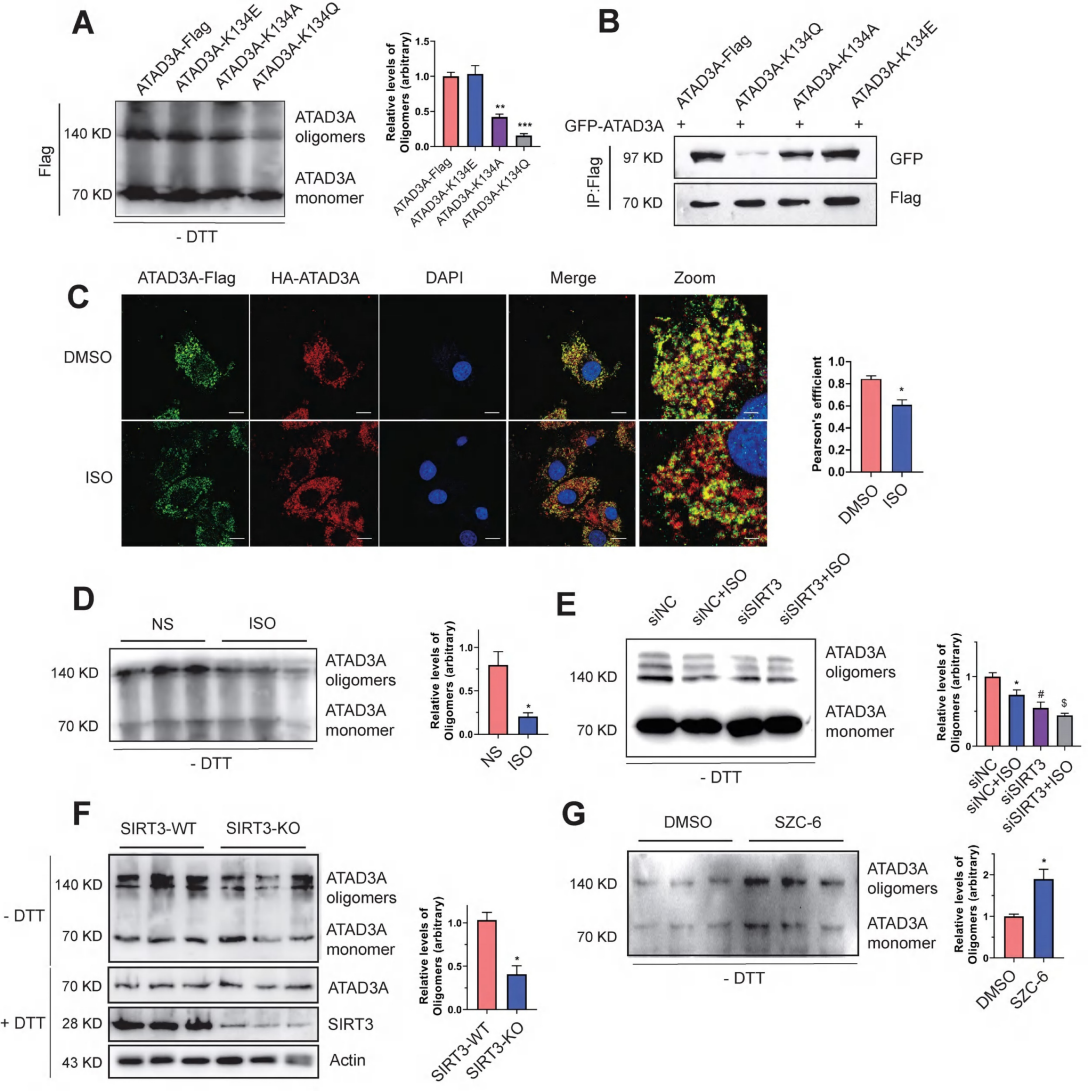
** Decreased ATAD3A oligomerization induced by SIRT3 downregulation*.*** (A) ATAD3A oligomers were analyzed in H9c2 cells transfected with ATAD3A-Flag and its different mutants (ATAD3A-K134Q, ATAD3A-K134A, ATAD3A-K134E). (B) H9c2 cells were transfected with vectors encoding GFP-ATAD3A and ATAD3A-Flag with its different mutants, followed by immunoprecipitation with anti‐Flag beads and immunoblot analysis with anti‐GFP. (C) Quantification of ATAD3A-Flag versus HA-ATAD3A colocalization by confocal immunofluorescence (scale bar = 20 μm) and magnified images (scale bar = 5 μm). (D) ATAD3A oligomers were analyzed in total lysates of cardiac tissue from ISO-treated mice. (E) ATAD3A oligomers were analyzed in NRCMs transfected with siSIRT3 or siNC, followed by incubation with ISO (10 μM, 24 h). (F) ATAD3A oligomers were analyzed in total lysates of cardiac tissue from SIRT3-WT and SIRT3-KO mice. (G) ATAD3A oligomers were analyzed in NRCMs treated with DMSO or SZC-6 (20 μM, 24 h). The data are presented as the mean ± SEM. ^*^P < 0.05, n = 3.

Because K134 lies within the oligomerization domain of ATAD3A, we wondered whether the K134 mutation affects ATAD3A oligomerization in cardiomyocytes. With the aim of simulating the acetylated state and neutralizing the positive charge, we substituted K134 with glutamine (K134Q) or alanine (K134A). To simulate deacetylated ATAD3A, the mutant K134E (lysine was switched for glutamic acid) was employed. The K134Q and K134E mutations were mostly utilized in our research. Native gel electrophoresis, a common method for protein oligomerization experiments, was used to evaluate ATAD3A oligomerization. Expression of the acetyl-deficient K134Q mutant diminished the ability of ATAD3A to oligomerize, as the level of the oligomers was significantly lower than that in WT-ATAD3A-expressing cells (Figure 3A). Coimmunoprecipitation (Co-IP) also indicated that the K134Q mutant was restricted in its ability to form an oligomer with GFP-ATAD3A, and the K134E mutant bound to GFP-ATAD3A more efficiently to form oligomers (Figure 3B). Acetylation of ATAD3A under ISO stimulation predicted that oligomeric levels of ATAD3A may be correspondingly reduced upon hypertrophic stimuli. Immunofluorescence assays suggested that colocalization of ATAD3A with different tags in cardiomyocytes under ISO stimulation was reduced (Figure 3C). Decreased levels of ATAD3A oligomer were also detected in ISO-stimulated myocardial tissue of rats (Figure 3D). We hypothesize that ISO negatively affects the deacetylation activity of SIRT3 and thus inhibits the oligomerization of ATAD3A. Compared to the siNC group, the decrease in ATAD3A oligomer in SIRT3 knockdown cardiomyocytes under ISO stimuli was more severe (Figure 3E). Additionally, the results showed decreased levels of ATAD3A oligomerization in the cardiac tissue of SIRT3-KO mice (Figure 3F). The protein levels of ATAD3A were unaltered in total protein extracts, excluding the possibility that enhanced ATAD3A oligomerization is due to increased its protein expression. We used SZC-6, a novel SIRT3 agonist that we have reported^44^, to activate SIRT3, and it significantly increased the oligomerization level of ATAD3A (Figure 3G). Thus, decreased ATAD3A oligomerization under cardiac hypertrophy may result from decreased SIRT3 activity. Interestingly, we also found that overexpression of ATAD3A in NRCMs significantly increased ATAD3A oligomers while reducing the acetylation proportion of ATAD3A (Figure S4).

### ATAD3A has mitochondrial protection and ROS scavenging effects

**Figure 4:**
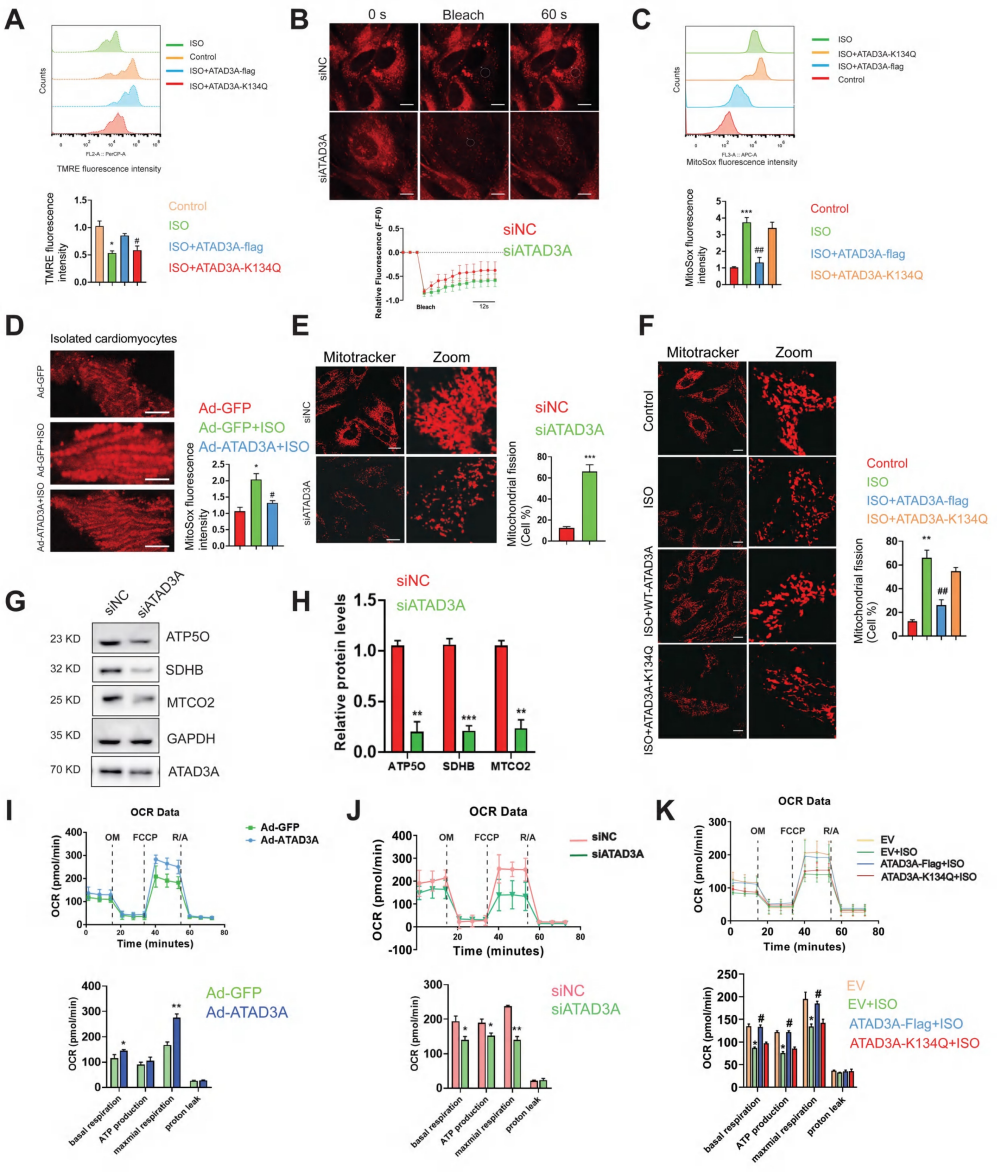
**ATAD3A promotes mitochondrial homeostasis and respiratory activity.** (A) Flow cytometric detection of TMRE-stained H9c2 cells and quantification of the mean TMRE signal. H9c2 cells were transfected with ATAD3A-Flag or ATAD3A-K134Q for 48 h, followed by incubation with ISO (10 μM, 24 h). (B) Representative confocal images of NRCMs stained with TMRE. The region of interest (as shown by the circled area) was irradiated with a 750 nm laser to achieve photobleaching. Quantification of mitochondrial dynamics using FRAP by measuring the recovery rate of TMRE fluorescence (scale bar = 10 μm). (C) Flow cytometric detection of MitoSoxRed-stained H9c2 cells and quantification of the mean signal. H9c2 cells were transfected with ATAD3A-Flag or ATAD3A-K134Q for 48 h, followed by incubation with ISO (10 μM for 24 h). (D) Representative confocal images showing MitoSoxRed fluorescence intensities in isolated rat adult cardiomyocytes. Cells were transfected with Ad-GFP or Ad-GFP and then exposed to ISO (scale bar = 5 μm). (E) NRCMs were transfected with siNC or siATAD3A for 48 h, followed by incubation with ISO (10 μM, 24 h). Mitochondrial fission was detected by MitoTracker Red with confocal microscopy (scale bar = 20 μm). (F) NRCMs were transfected with ATAD3A-Flag or ATAD3A-K134Q for 48 h followed by incubation with ISO (10 μM, 24 h). Mitochondrial fission was detected by MitoTracker Red with confocal microscopy (scale bar = 20 μm).(G, H) Representative immunoblot of ATP5O, SDHB and MTCO2 in NRCMs transfected with siNC or siATAD3A for 48 h. Quantitation of the WB results is shown. (I) Seahorse assay for the OCR in H9c2 cells with or without Ad-ATAD3A infection. Quantification of basal respiration, ATP production, maximal respiration, spare capacity and proton leak is shown. (J) Seahorse assay for the OCR in H9c2 cells. Cells were transfected with siNC or siATAD3A followed by incubation with ISO (10 μM, 24 h). Calculations of basal respiration, ATP production, maximal respiration, and spare respiratory capacity are shown. (K) Seahorse assay for the OCR in H9c2 cells. Cells were transfected with ATAD3A-Flag or ATAD3A-K134Q followed by incubation with ISO (10 μM for 24 h). Calculations of basal respiration, production, maximal respiration, and spare respiratory capacity are shown. The data are presented as the mean ± SEM. ^*^P < 0.05,^ #^*P* < 0.05, n = 3.

Whether ATAD3A could protect mitochondria from excessive mitochondrial damage upon hypertrophic stimuli was then investigated. In ISO-treated cardiomyocytes, most mitochondria were not labeled by TMRE, reflecting their electrochemically inactive status (Figure 4A). Mitochondria with WT-ATAD3A displayed increased TMRE staining. Cardiomyocytes expressing ATAD3A-K134Q exhibited lower TMRE intensity than cardiomyocytes expressing WT-ATAD3A, which indicated marked mitochondrial depolarization. Fluorescence recovery after photobleaching (FRAP) was utilized to evaluate mitochondrial functional connectivity. NRCMs were labeled with TMRE, and then chosen areas of the mitochondrial network were photobleached. Throughout time, the FRAP signal was quantified. In ATAD3A-depleted NRCMs, the rate of recovery and maximal fluorescence recovery were lower than those in controls (Figure 4B), indicating impaired mitochondrial connection. MitoSOX measurement indicated much lower MitoSOX fluorescence in WT-ATAD3A-expressing cells, the effect of which was attenuated by ATAD3A-K134Q (Figure 4C). In adult mouse cardiomyocytes, those with ATAD3A-expressing adenovirus infection had lower mitochondrial superoxide generation (Figure 4D). Mitochondria in the siNC groups retained typical fiber-like structures. In contrast, upon treatment with si-ATAD3A, the mitochondria appeared punctate, indicating mitochondrial fragmentation (Figure 4E). Mitochondria in WT-ATAD3A-expressing cardiomyocytes, however, remained normal after ISO challenge (Figure 4F), demonstrating that ISO-induced mitochondrial fragmentation could be prevented by forced WT-ATAD3A expression but not ATAD3A-K134Q. We used an extracellular flux analyzer (Seahorse XF96) to measure oxygen consumption rates (OCRs) in H9c2 cells to evaluate mitochondrial respiratory activity. Increased mitochondrial function in the Ad-ATAD3A group compared with the Ad-GFP group (Figure 4I). In contrast, inhibition of ATAD3A decreased mitochondrial respiratory activity (Figure 4J), which is consistent with the decreased expression of respiratory chain complex components (Figure 4G and Figure 4H). The cells expressing WT-ATAD3A, but not ATAD3A-K134Q, displayed increased basal respiration, ATP production, and maximal respiration compared to the ISO group (Figure 4K). These findings demonstrate that ATAD3A protects against mitochondrial ROS, which is associated with protecting mitochondrial integrity.

### Upregulation of ATAD3A alleviates pathological hypertrophy induced by ISO

**Figure 5:**
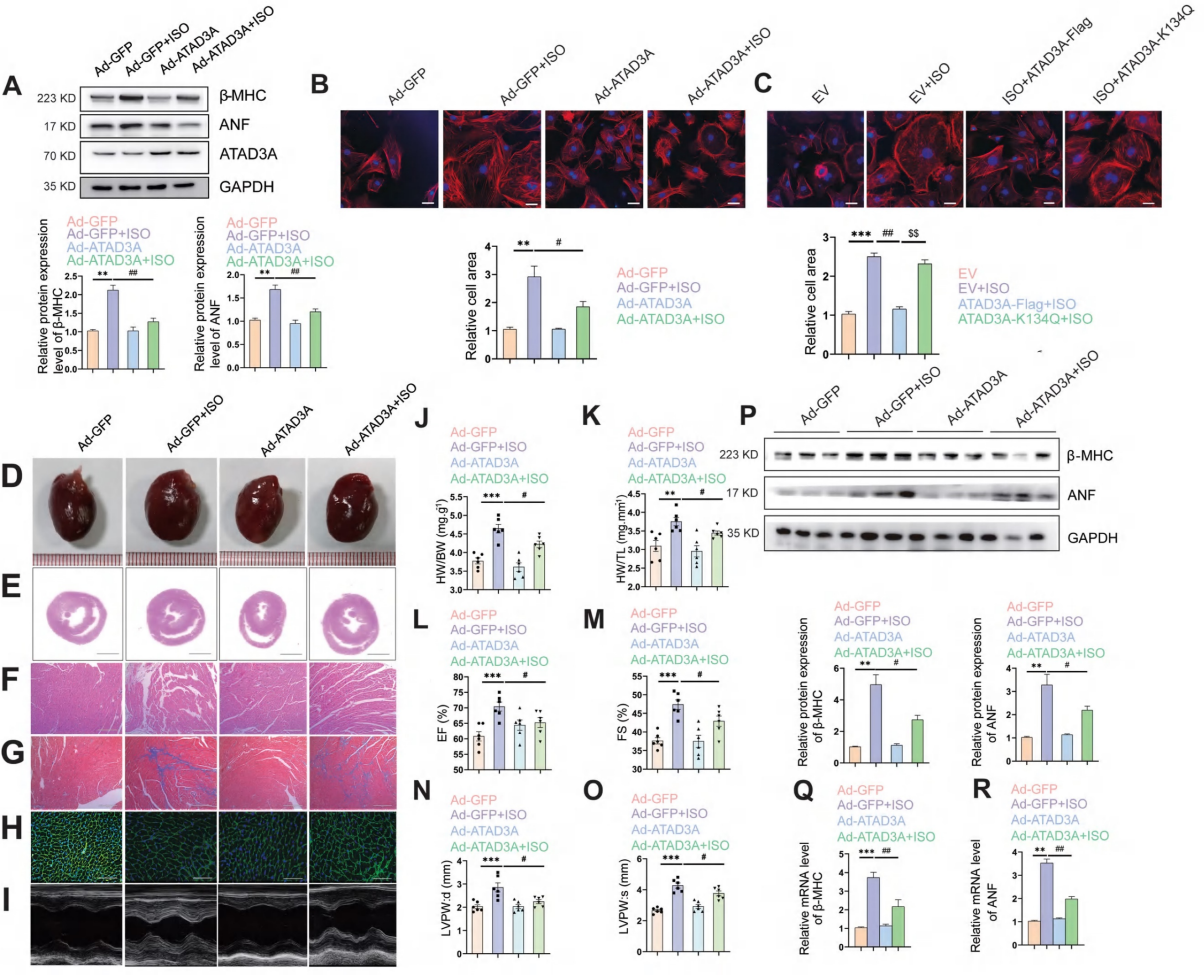
** Overexpression of ATAD3A attenuates ISO-induced cardiac hypertrophy.** Myocardial injection of recombinant adenovirus (Ad-ATAD3A or Ad-GFP) was performed in SD rats, and then rats were subjected to injections of ISO with normal saline (NS) for one week. Pathological changes in the myocardium were observed. (A) NRCMs were treated with with Ad-GFP or Ad-ATAD3A for 48 h, followed by incubation with ISO (10 μM, 24 h). The protein expression of ANF, β-MHC and ATAD3A was detected by western blot analysis. (B, C) The surface area of NRCMs was measured (Scale bar = 20 μm). (D) Gross morphologic examination. (E, F) H&E staining (E, scale bar = 5 mm; F, scale bar = 200 μm). (G) Heart sections stained with Masson's trichrome to detect fibrosis (scale bar = 200 μm). (H) WGA staining (scale bar= 50 μm). (I) Echocardiography. (J, K) The HW/BW ratio and HW/TL ratio were calculated. (L-O) Echocardiographic parameters were measured. (P-R) The protein and mRNA levels of β-MHC and ANF in cardiac tissues were measured by Western blot analysis and qRT‒PCR. The data are presented as the mean ± SEM. ^**^P < 0.01, ^***^P < 0.001,^ #^*P* < 0.05, ^##^*P* < 0.01.

Since ATAD3A significantly inhibits mitochondrial oxidative stress and bioenergetics impairment (Figure 4C-K), we explored whether ATAD3A attenuates ISO-induced cardiac hypertrophy. Forced expression of ATAD3A by transfection with WT-ATAD3A decreased the protein levels of hypertrophic markers, including β-MHC and ANF (Figure 5A), and markedly decreased the cell surface area in NRCMs (Figure 5B). However, ATAD3A-K134Q did not exert a protective effect (Figure 5C). To further confirm the anti-hypertrophic effect of ATAD3A *in vivo*, rats had their left ventricles transduced with either a recombinant adenovirus of ATAD3A (Ad-ATAD3A) or a GFP control (Ad-GFP) before being exposed to ISO. As the HW/BW ratio and echocardiographic parameters showed, overexpression of ATAD3A alleviated ISO-induced cardiac injury, as indicated by improved morphological changes, a decrease in the disorganized myocardium, decreased cell size and extracellular matrix, and decreased fibrosis (Figure 5D-O). In cardiac tissues, the protein and mRNA levels of hypertrophic indicators were evaluated. ATAD3A overexpression substantially reduced the expression of ANF and β-MHC (Figure 5P-R). These findings demonstrate that the overexpression of ATAD3A is sufficient to suppress cardiac hypertrophy and that the acetylation status of ATAD3A influences this process.

### ATAD3A interplay with MAM protein

**Figure 6:**
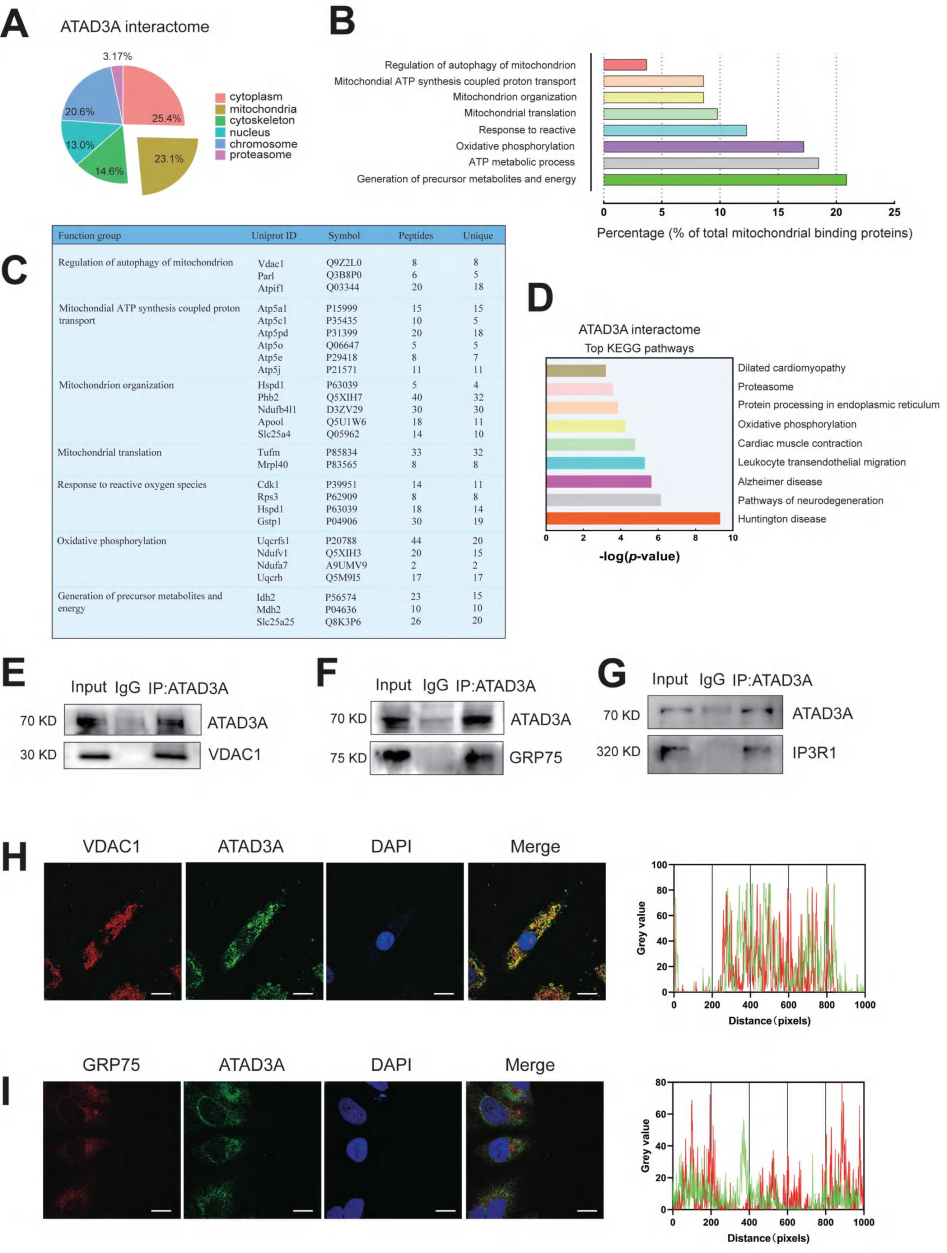
** ATAD3A binds to the IP3R1-GRP75-VDAC1 complex in the heart.** (A) Cellular location of ATAD3A interactors in the heart tissue of mice with cardiac hypertrophy. (B) Among mitochondrial candidates, the generation of precursor metabolites and energy accounts for up to over 20% of all mitochondrial candidates. (C) Gene Ontology (GO) molecular function enrichment analysis was performed on ATAD3A binding protein in mitochondria. (D) Top 9 KEGG pathways enriched by analysis of the ATAD3A interactome. (E) Co-IP of ATAD3A-VDAC1 in cardiomyocytes, immunoprecipitated and revealed using the indicated antibodies. (F) Co-IP of ATAD3A-GRP75 in cardiomyocytes, immunoprecipitated and revealed using the indicated antibodies. (G) Co-IP of ATAD3A-IP3R1 in cardiomyocytes, immunoprecipitated and revealed using the indicated antibodies. (H) VDAC1 and ATAD3A colocalization was shown by two-color immunofluorescence staining in cardiomyocytes (scale bar = 20 μm). Gray value analysis of the VDAC1 and ATAD3A signals is shown. (I) GRP75 and ATAD3A colocalization shown by two-color immunofluorescence staining in cardiomyocytes (scale bar = 20 μm). A gray value analysis of the GRP75 and ATAD3A signals is shown.

It has been shown that the oligomerization of proteins can affect the diversity and specificity of biochemical pathways and is associated with regulating enzyme activities, cooperativity and stability. Since ATAD3A can form oligomers and has been reported to form heterodimers with ATAD3B to affect mitochondrial autophagy 45, it seems unlikely that ATAD3A regulates mitochondrial homeostasis alone. We hypothesized that ATAD3A can interact with other mitochondrial proteins and ultimately alter the function of mitochondria. Using immunoprecipitation-mass spectrometry (IP-MS) analysis, we sought to discover protein candidates in rat heart tissue that interact with ATAD3A. After affinity purification, tandem mass spectrometry analysis identified 604 proteins presumed to be linked to ATAD3A. These proteins are engaged in numerous cellular function pathways (Figure 6A). Concentrating on the mitochondrial protein candidates, we identified an enrichment of proteins involved in mitochondrial nucleoid assembly and energy generation (Figure 6B). We highlighted the top proteins that interacted with ATAD3A, screening from "Regulation of autophagy of mitochondrion" and "Generation of precursor metabolites and energy" pathways through GO biological process analysis (Figure 6C). KEGG pathway analysis showed that ATAD3A was involved in regulating multiple pathways (Figure 6D). We found some proteins localized in the MAM structure, which suggested that ATAD3A might regulate the MAM structure in the heart. The interaction between ATAD3A and IP3R1, GRP75 and VDAC1 was confirmed by a Co-IP assay in cardiomyocytes (Figure 6E-G). Moreover, immunofluorescence staining demonstrated that ATAD3A colocalized with GRP75 and VDAC1 (Figure 6H and Figure 6I). Since the IP3R1-GRP75-VDAC1 complex is a crucial regulator of MAMs, it is most likely that ATAD3A can interact with the IP3R1-GRP75-VDAC1 complex.

### SIRT3-ATAD3A axis regulates MAM formation homeostasis

**Figure 7:**
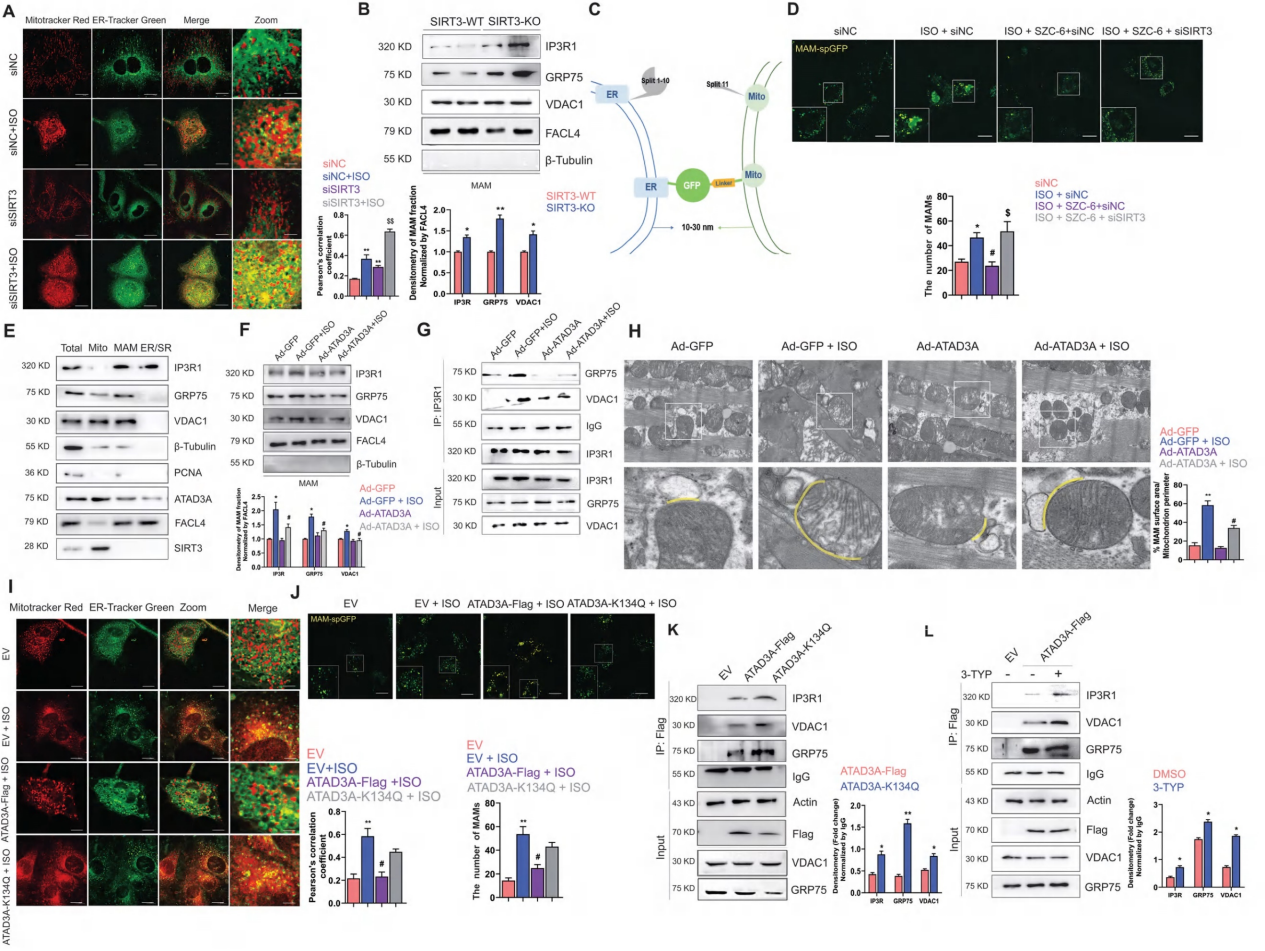
**ATAD3A inhibits ISO-induced excessive MAM formation.** (A) Colocalization of mitochondria and ER. Mitochondria were labeled with MitoTracker Red; ER was labeled with ER-Tracker Green. A representative confocal microscopy image is shown (scale bar =10 μM). The colocalization of mitochondria and ER was determined by calculation of the Pearson correlation coefficient. (B) Immunoblot analysis of the MAM fractions isolated from cardiac tissue of SIRT3-WT and SIRT3-WT mice. (C) Design of a reporter of mitochondria-ER contacts. When the distance between mitochondria and the ER is approximately 10-30 nm, spGFP1-10 and spGFP11 will refold and emit green fluorescence. (D) Quantification of the MAMs was counted and quantified in at least 30 randomly picked cells (scale bar = 20 μm). (E) Immunoblot analysis of subcellular fractions isolated from cardiac tissue of SIRT3-WT mice. (F) After myocardial transduction with Ad-GFP or Ad-ATAD3A virus, rats were subjected to injections of ISO or normal saline (NS) for one week. Heart tissue was collected. (G) After myocardial transduction with Ad-GFP or Ad-ATAD3A virus, SD rats were subjected to injections of ISO or normal saline (NS) for one week. Lysates of heart tissue were subjected to IP using an anti-IP3R1 antibody followed by WB analysis using specific antibodies. The percentage of MAM surface area per mitochondrion perimeter in each microscopic field is shown. (H) Representative TEM images showing the association of SR/ER and mitochondria in recombinant virus-injected ventricular wall tissue from rats. Inset: yellow dotted line indicating the surface area of MAM (scale bar =1 μm). (I) Mitochondria were labeled with MitoTracker Red, and the ER was labeled with ER-Tracker Green. A representative confocal microscopy image is shown (scale bar =10 μM). (J) Quantification of the MAMs was counted and quantified in at least 30 randomly picked cells (scale bar = 20 μm). (K) H9c2 cells were transfected with ATAD3A-Flag or ATAD3A-K134Q. Cells were subjected to IP with anti-Flag antibody followed by western blotting. (L) H9c2 cells expressing ATAD3A-Flag were treated with 3-TYP or DMSO and then subjected to IP with an anti-Flag antibody followed by Western blotting. The data are presented as the mean ± SEM. ^*^P < 0.05, ^**^P < 0.01, ^#^*P* < 0.05, ^##^*P* < 0.01, ^$$^*P* < 0.01, n = 4. EV: empty vector.

Our previous work pointed out that SIRT3 agonists could relieve oxidative stress and improve mitochondrial structural integrity, thus alleviating cardiac hypertrophy 46-48. In addition, SIRT3 has been reported to be an integral regulator of ER function. However, the role of SIRT3 in regulating mitochondria and ER crosstalk remains unclear. Here, we were the first to report that knockdown of SIRT3 leads to increased colocalization of mitochondria and endoplasmic reticulum and a further significant increase in colocalization with ISO stimulation (Figure 7A).

An increase in MAM formation was detected in the heart tissue of SIRT3-KO mice, as shown by immunoblot analysis of the IP3R1-GRP75-VDAC1 complex (Figure 7B). A new technique was kindly provided by Tong and used to detect MAM formation 49. It is a luminous split protein system, with one portion lying in the endoplasmic reticulum (ER) and the other in the mitochondria. Only until mitochondria and the endoplasmic reticulum (ER) are close enough will these two components generate a functional and full protein, spGFP. Hence, the GFP puncta mark the MAMs within the cell (Figure 7C). The ISO-induced increase in MAM-spGFP puncta in NRCMs was reversed by SZC-6, an agonist of SIRT3 (Figure 7D). The presence of the IP3R1-GRP75-VDAC1 complex at the MAMs was then confirmed. In subcellular fractions extracted from SIRT3-WT mice heart tissue, we detected the expression of ATAD3A in the MAM fraction (Figure 7E). Interestingly, overexpression of ATAD3A reversed the ISO-induced increase in the IP3R1-GRP75-VDAC1 complex in the MAMs of rat ventricular wall tissue. The aberrant connection generated by ISO between these proteins at the MAM interface is diminished. In conjunction with the results we observed in the TEM assay, MAM formation in ATAD3A-expressing NRCMs was not significantly increased in response to ISO stimulation (Figure 7F-H). However, MAMs were significantly induced in ATAD3A-K134Q-expressing NRCMs (Figure 7I and Figure 7J), along with increased interactions between ATAD3A-K134Q and IP3R1-GRP75-VDAC1 (Figure 7K). We investigated whether hyperacetylated ATAD3A interacts abnormally with components of the IP3R1 channel complex. Treatment with 3-TYP increases the interaction between ATAD3A and IP3R1, VDAC1 and GRP75. (Figure 7L). Taken together, these findings suggest that ATAD3A plays an important role in regulating MAM formation by regulating the IP3R1-GRP75-VDAC1 complex in cardiac tissue, and this protective effect against hypertrophic stimuli is regulated by the deacetylating activity of SIRT3.

### ATAD3A prevents MAM-associated mitochondrial calcium overload

**Figure 8:**
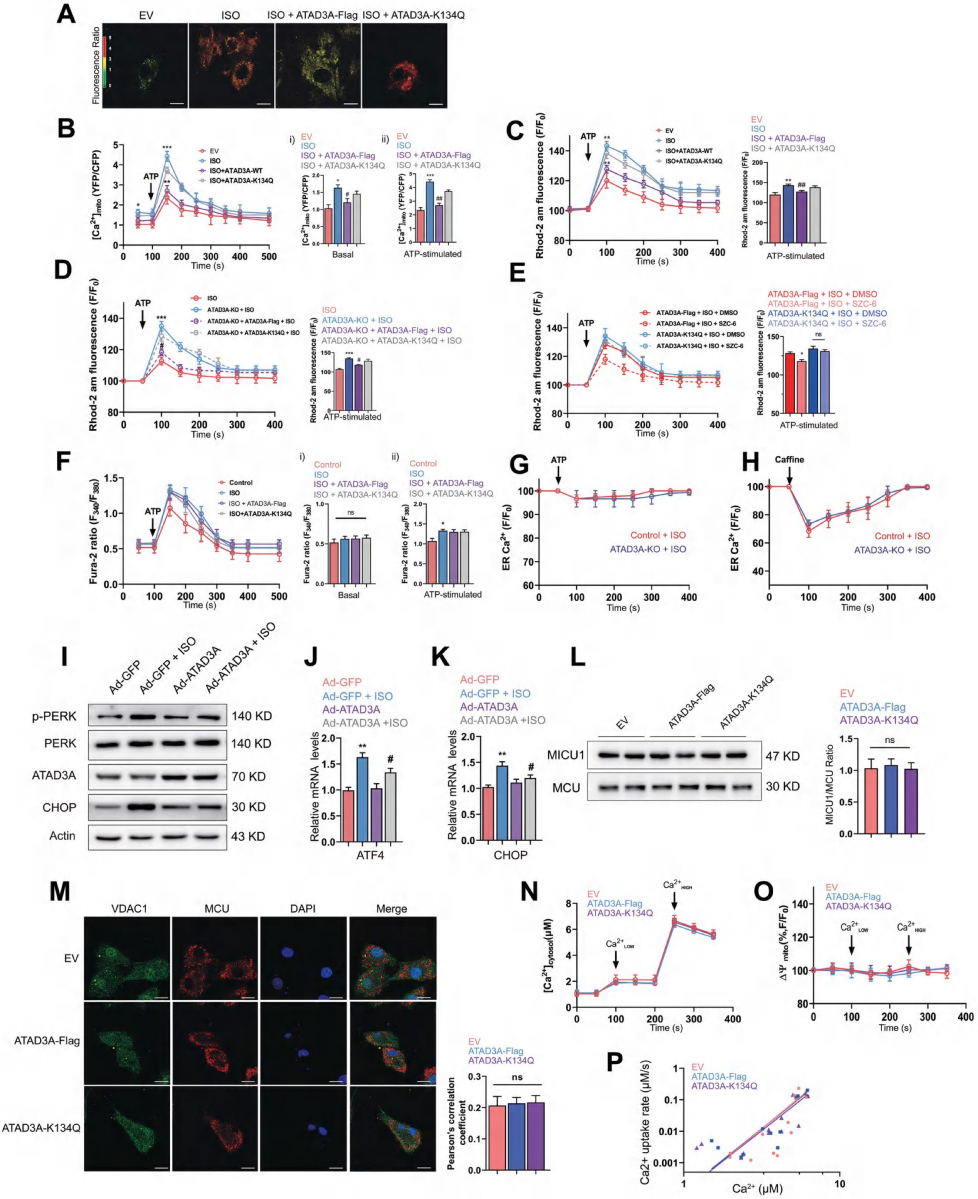
** ATAD3A regulates calcium transfer to mitochondria.** (A, B) Mitochondrial calcium levels were assessed in NRCMs using a mitochondrial-targeted cameleon CFP/YFP FRET probe. Cells were transfected with an empty vector (EV), ATAD3A-Flag or ATAD3A-K134Q. Representative photomicrographs are shown (scale bar = 10 μm). The spectrum color scale represents the ratio of emitted fluorescence (YFP/CFP). Basal [Calcium]_mito_ (Bi) and [Calcium]_mito_ after stimulation with 100 μmol/l ATP in H9c2 cells are shown (Bii). (C-E) Mitochondrial Calcium flux was measured using Rhod2-AM. [Calcium]_mito_ after stimulation with 500 nmol/L ATP is shown. (F) Representative Fura-2 ratio fluorescence tracing from cardiomyocytes. The arrow indicates the timing of ATP addition. Basal [Calcium]_c_ (Fi) and [Calcium]_c_ after stimulation with 100 μmol/l ATP in H9c2 cells are shown (Fii). (G, H) Changes in ER calcium levels measured with an ER-targeted probe in WT and ATAD3A-KO H9c2 cells after the addition of ATP (100 μmol/l, G) or caffeine (10 mmol/l, H) n = 4 (with > 20 cells per individual experiment). (I) The protein levels of p-PERK, PERK, CHOP and ATAD3A in cardiac tissues were measured by Western blot analysis. (J, K) The mRNA levels of ATF4 and CHOP in cardiac tissues were measured by qRT‒PCR. (L) Detection of MICU1 and MCU by immunoblotting in isolated pure mitochondria from H9c2 cells. The right panel displays the MICU1 to MCU ratio densitometric analysis normalized to an empty vector group (EV). (M) Representative immunofluorescence colocalization of MCU with VDAC1 in NRCMs (scale bar = 20 μm). The data are presented as the mean ± SEM. (N) Time course of the mitochondrial clearance of the [Calcium]_c_ rise, measured by Fura2, upon addition of CaCl^2^ bolus (10 µM followed by 50 µM) in suspensions of mitochondria of H9c2. (O) Recordings of mitochondrial membrane potential, measured by TMRE. (P) Double logarithmic plot of the initial Calcium uptake rates against the measured peak [Calcium]_c_ in mitochondria of H9c2 cells (n = 4). The slope of each linear ft is indicated.

Previous studies have demonstrated that alterations in MAM composition affect calcium homeostasis 50, 51. Therefore, the regulation of MAM homeostasis by ATAD3A may allow it to exert protective effects on calcium transfer between the two organelles. IP3R1-mediated calcium release from ER stores was induced by ATP, and mitochondrial, cytosolic, and ER calcium levels in cardiomyocytes were then assessed. WT-ATAD3A overexpression lowered basal [Ca^2+^]_mito_ levels compared with ISO treatment, but ATAD3A-K134Q did not (Figure 8A-B); meanwhile, excess mitochondrial calcium was detected in ATAD3A KO cells (Figure 8C). However, 2-APB treatment prevented calcium overload in ATAD3A KO cells, which suggests that ATAD3A mainly regulates IP3R1-mediated mitochondrial calcium inflows (Figure S3). In addition, the reintroduction of WT-ATAD3A restored mitochondrial calcium levels in ATAD3A KO cells to lower levels. In contrast, overexpression of ATAD3A-K134Q failed to return mitochondrial calcium to normal levels (Figure 8D). Similarly, SZC-6 reduced mitochondrial calcium only in ATAD3A-Flag-expressing H9c2 cells (Figure 8E). Thus, the acetylation level of ATAD3A affects its regulation of mitochondrial calcium. Using Fura-2, a cytosolic calcium reporter, we also monitored [calcium]_c_ and discovered a larger peak of [calcium]_c_ in response to ATP stimulation upon administration of ISO. WT-ATAD3A and ATAD3A-K134Q overexpression did not significantly affect cytoplasmic calcium levels (Figure 8F). Additionally, we measured [calcium]_ER_ levels using the ER-specific calcium reporter Fluo-4 am. The baseline and ATP-induced ER calcium depletion rates were comparable across all groups, indicating that ATAD3A KO had no effect on the ER calcium storage or release machinery (Figure 8G). In cardiomyocytes, calcium release from the ER/SR is facilitated in part by ryanodine receptors and IP3R1-mediated release. To assess the effect of ATAD3A on ryanodine receptor-mediated calcium flow, we compared the response of cardiomyocytes to caffeine (calcium release via ryanodine receptors). ATAD3A did not affect the increase in cytoplasmic calcium flow due to ryanodine receptor activation (Figure 8H). ATAD3A overexpression could also attenuate ISO-induced ER stress. Markedly decreased PERK phosphorylation and CHOP expression were observed in heart tissues overexpressing ATAD3A (Figure 8I). ATAD3A significantly reduced the mRNA levels of CHOP and ATF4 compared to the ISO group (Figure 8J and Figure 8K). It is commonly believed that the mitochondrial calcium uniporter (MCU) is the central mechanism for calcium uptake into mitochondria. The mitochondrial membrane potential, which is maintained by proton extrusion, is the driving force behind calcium uptake. We questioned whether ATAD3A may impact the mitochondrial calcium uniporter directly. Examining the protein levels of the pore-forming protein MCU and its primary regulator MICU1 is critical since the ratio of these two factors currently governs the function of the mitochondrial calcium uniporter. In isolated mitochondria, neither ATAD3A-Flag nor ATAD3A-K134Q affected MICU1/MCU protein expression (Figure 8L), and the colocalization of VDAC1 and MCU was identical (Figure 8M). Using thapsigargin and CGP37157, inhibitors of the sarco/endoplasmic reticulum calcium-ATPase and the mitochondrial Na^+^/Ca^2+^ exchanger, direct mitochondrial calcium absorption via the mitochondrial calcium uniporter was examined. The same rate of mitochondrial calcium clearance was recorded in the mitochondria of each treatment group (Figure 8N). Notably, each group had the same amount of mitochondrial polarization, which indicated that the mitochondrial driving force was not inhibiting calcium uptake (Figure 8O). A double logarithmic plot of the initial calcium uptake rates against different [Ca^2+^]_c_ was used to determine how much mitochondrial uptake depends on [Ca^2+^]_c_. Since the slopes of the linear ft were the same, neither the activation threshold nor the cooperative activation changed (Figure 8P). Thus, ATAD3A had no effect on the structure or function of the heart's mitochondrial calcium uniporter. This demonstrates that the decreased mitochondrial calcium uptake in ATAD3A-expressing cardiomyocytes is mostly attributable to the diminished functional calcium coupling between the reticulum and mitochondria. Together, ATAD3A inhibits the abnormal generation of MAMs upon hypertrophic stimuli, and the excess calcium flux in MAM microdomains is then suppressed, which preserves mitochondrial calcium transport homeostasis and reduces mitochondrial damage and endoplasmic reticulum stress in the myocardium.

## Discussion

Our analysis of the ATAD3A interactome indicated that ATAD3A significantly affects the generation of precursor metabolites and energy, ATP metabolic process, oxidative phosphorylation, response to ROS and mitochondrial translation of the heart. This is the first report that reveals the protective effect of ATAD3A on the homeostasis of mitochondrial calcium and MAM formation in hypertrophic hearts. An unexplored posttranslational modification of ATAD3A, acetylation, is critical for the function of ATAD3A in protecting mitochondria from harm in response to hypertrophic stimulation.

ATAD3A was first identified from mitochondrial nucleoprotein complexes in rat liver 52. Since the mitochondrial ribosome is the main site to which ATAD3A binds, changes in ATAD3A expression affect the maintenance and replication of mtDNA 53. It was not surprising to observe that the deletion of ATAD3A reduced capacity of aerobic mitochondrial respiration, as ATAD3A expression level of mitochondrial complexes. Consensus on a secondary structure prediction, the ATAD3A protein has a core transmembrane segment (TMS) that binds the protein in the inner membrane and a C-terminal domain in the matrix, allowing it to participate in dynamic interactions between the outer and inner membranes 54. Correspondingly, ATAD3A-expressing cells exhibited a lower level of mitochondrial fragmentation and increased mitochondrial connectivity. Transmembrane proteins such as ATAD3A might interact with various functional proteins in signal transduction 55-57. However, the N-terminal domain (amino acids 44-247) of ATAD3A previously proposed to bind to the mtDNA D-loop is directed away from the mitochondrial matrix 58. Meanwhile, a back-titration ELISA measurement and immunofluorescence analysis on freshly purified human mitochondria showed that the N-terminal region of ATAD3A is outside of the inner membrane 59. As our truncation experiment showed, the N-terminus of ATAD3A mainly interacts with SIRT3.

Among the predicted acetylation sites on ATAD3A, K134 is deacetylated by SIRT3 because of its location at the interface of two CCs. K134Q may form a salt bridge with glutamate 142 (E142) in another CC to stabilize the intermolecular oligomer. The coiled-coil (CC) domain serves as a classic oligomerization domain for a wide variety of proteins, including structural proteins and transcription factors 60. It was reported that the CC domain of ATAD3A is required for ATAD3A oligomerization, as deletion of the CC domain abrogated ATADA3A oligomerization even when the ATPase domain was present 61. However, Seiji et al. recently reported that σ1R retains ATAD3A as a monomer to induce MAM formation 62. In our experiment, highly acetylated ATAD3A may be more inclined to be a monomer. The decreased oligomer-to-monomer ratio might contribute to MAM formation, which is reversed by the upregulation of ATAD3A.

ER and mitochondria crosstalk via MAMs is believed to be critical in maintaining cellular homeostasis 63. An imbalance in MAMs is involved in the development and progression of chronic heart failure 64, 65. Moreover, the IP3R1-GRP75-VDAC1 complex has been proposed to serve a regulatory function in MAMs, silencing the GRP75 can inhibit ER-mitochondrial calcium transport, reduce mitochondrial oxidative stress, and thereby prevent atrial remodeling caused by diabetes 66. It was also reported that IP3R inhibitor, Xestospongin C, abolished the IP3R-induced Ca^2+^ responses, and significantly suppressed cardiomyocyte hypertrophy 67. Sustained mitochondrial calcium overload-mediated cardiomyocyte apoptosis will eventually lead to severe myocardial structural remodeling. IP3Rs can affect the apoptosis of cardiomyocytes by affecting the opening and closing of MPTP. Studies have reported that IP3R1 is highly expressed in the myocardium. Silencing IP3R1 can reduce heart injury, reduce calcium overload, and reduce the risk of heart failure in MI/R rats 68. The development of cardiac hypertrophy is also accompanied by electrophysiological remodeling. IP3R1-GRP75-VDAC1-mediated mitochondrial calcium flow is involved in the excitation-contraction coupling process of the myocardium 69. Here, we found that ATAD3A is localized in the MAM and binds to the IP3R1-GRP75-VDAC1 complex. The binding of IP3R1 to VDAC1 was significantly increased in response to ISO stimulation. Interestingly, overexpression of ATAD3A decreased the integrity of the IP3R1-GRP75-VDAC1 complex. It is well known that the IP3R1 complex constitutes the most crucial channel for calcium flux within the MAM micro-domain, which mediates ERS-mitochondrial oxidative stress in diabetic atrial remodeling 70. The acetylation status of ATAD3A affects its control of MAM homeostasis. Correspondingly, the knockdown of SIRT3 drives an abnormal increase in MAM formation. Our results demonstrated the regulation of MAM formation by the SIRT3-ATAD3A axis. Although ATAD3A's target substrates have not been identified, we hypothesize that ATAD3A may also be able to impact the phosphorylation levels of these interacting partners in MAM, a concept that merits further exploration.

The mitochondria's strategic localization close to ER/SR calcium channels makes it possible to decode calcium signals. Recent reports in cardiomyocytes indicated increased MAMs in db/db mice and a type 1 diabetic animal model 71. Both models with muscular systolic dysfunction show increased mitochondrial calcium uptake. MAM may be protective in early HF development. However, the continued formation of MAM leads to a sustained increase in mitochondrial calcium levels, leading to increased mitochondrial ROS production and mitochondrial dysfunction. Mitochondrial ROS can also trigger ER stress. Mitochondrial calcium signaling generates the majority of the energy essential for cardiomyocyte function by activating calcium-sensitive matrix dehydrogenases and subsequent mitochondrial oxidative phosphorylation-driven ATP synthesis. ATAD3A prevents ISO-induced mitochondrial calcium accumulation and ROS generation, improving mitochondrial dysfunction and ER stress via suppression of MAM formation. ATAD3A rescued ISO-induced mitochondrial calcium mishandling without detecting changes in reticular calcium stocks, cytosolic calcium transients, and calcium currents at the plasma membrane. Thus, we speculate that ATAD3A mainly regulates IP3R1-mediated calcium release from the SR/ER. Whereas mitochondrial calcium uptake is a two-step process, cytosolic calcium is first carried into the mitochondrial outer membrane by VDAC1 and subsequently into the matrix by the mitochondrial calcium uniporter (MCU). While our findings support that abnormal MAM generation and IP3R1-mediated calcium transfer trigger mitochondrial dysfunction in ATAD3A-deficient cells, the absence of a substantial role for MCU raises an intriguing argument. In fact, MCU is known to mediate HF under situations of mitochondrial malfunction and ER stress.

However, we found that neither WT-ATAD3A nor ATAD3A-K134Q overexpression altered the structure and function of the mitochondrial calcium uniporter in cardiomyocytes. This finding implies that MCU-dependent calcium influx into mitochondria does not contribute to ATAD3A deficiency-induced mitochondrial dysfunction. SIRT3 deficiency can induce exaggerated MCU-mediated mitochondrial calcium uptake. We observed that SZC-6, a SIRT3 agonist, suppressed the activation of MCU, which is consistent with previous studies on the effect of SIRT3 on MCU.

According to our findings, ATAD3A dramatically alters cardiac mitochondrial respiration and ROS generation, which may have long-term ramifications for ventricular systolic function and pathology. These findings are consistent with the increasing amount of evidence that the ER and mitochondria interact and represent a crucial biological target underlying cardiac remodeling. Several studies have shown that MAMs are crucial for metabolic control 72. It has been observed that their dysfunction is connected with metabolic syndrome, including insulin signaling downregulation and hastened development of hyperlipidemia, obesity, and hypertension. Given the crucial relationship between mitochondrial function and cardiac health, ATAD3A-mediated mitochondrial energy supply, mitochondrial morphological homeostasis and mitochondrial antioxidant capacity could be essential mechanisms that drive cardiovascular disease susceptibility. We found that the ATAD3A monomer promotes the excessive development of the IP3R1-GRP75-VDAC1 complex, which ultimately results in the dysregulation of calcium handling and the disruption of mitochondrial homeostasis, despite its ability to be independent of changes in MCU activity. The fact that the SIRT3-ATAD3A axis had positive effects on mitochondrial parameters under the stimulation of pathological cardiac hypertrophy is encouraging and provides support for ATAD3A as a therapeutic target in future research.

## Conclusions

SIRT3 binds and deacetylates ATAD3A and K134, increasing the oligomerization level of ATAD3A. Downregulation of SIRT3 promoted the interaction between mitochondria and the ER and facilitated the excessive formation of MAMs. The IP3R1-GRP75-VDAC1 complex mediates ATAD3A hyperacetylation-induced calcium overload. ATAD3A attenuates mitochondrial oxidative stress and calcium overload, increases the Δψm and mitochondrial oxygen consumption, and protects cardiomyocytes from hypertrophic stimuli.

## Materials and Methods

All reagents, instruments and experimental procedures are introduced in detail in the supporting information.

## Supplementary Material

Supplementary figures and tables.

## Abbreviations

ANF: atrial natriuretic factor
β-MHC: myosin heavy chain β
CAT: catalase
DHE: dihydroethidium
Drp1: dynamin-related protein 1
Mfn2: mitofusin 2
MMP: mitochondrial membrane potential
NRCMs: neonatal rat cardiomyocytes
ISO: isoproterenol
TEM: transmission electron microscopy
3-TYP: 3-(1H-1,2,3-triazol-4-yl) pyridine
MAM: mitochondria-associated ER membrane
ER: endoplasmic reticulum
GRP75: glucose-regulated protein 75
IP3R: inositol 1,4,5-trisphosphate receptor
VDAC: voltage-dependent anion channel
ATAD3A: ATPase family AAA-domain containing protein 3A
