# Supplementary material for: The SIRT3-ATAD3A axis regulates MAM dynamics and mitochondrial calcium homeostasis in cardiac hypertrophy
Source: Int J Biol Sci. 2024 Jan 1;20(3):831–47. doi: 10.7150/ijbs.89253 (PMC10797690; doi:10.7150/ijbs.89253)
Supplement: Supplementary file 1 — Supplementary figures and tables. [file ijbsv20p0831s1.pdf]

SUPPORTING INFORMATION

Supplementary Information

SUPPORTING INFORMATION

The SIRT3-ATAD3A axis regulates MAM dynamics and mitochondrial calcium homeostasis in cardiac hypertrophy

Li ZY[1,&], Hu O[2,&], Xu SW[3], Lin CJ[1], Yu WJ[1], Ma DH[1], Lu J[1,*], Liu PQ[1,*]

Running title: The SIRT3-ATAD3A axis is involved in pathological cardiac hypertrophy.

This Manuscript Word document includes:

All reagents, instruments and experiment procedures

Supplementary Table S1 to S2

Supplementary FIGURE S1 to S5

RAW WB IMAGES

Study Animal

Jackson Laboratory provided male SIRT3-knockout (129-SIRT3tm1.1Fwa/J) mice and their wild-type (129/SvlmJ) controls (Bar Harbor, ME, USA). The Guangzhou University of Chinese Medicine Experimental Animal Center provided SD rats (male, 9-12 weeks) (Guangzhou, China). Sun Yat-sen University's Research Ethics Committee approved all surgical and care procedures, and they followed the Guide for the Care and Use of Laboratory Animals (NIH Publication No. 85-23, revised 1996). Both the rat and mice animal models were maintained in individually ventilated cages with 12 h daylight/dark cycles at 21-23 °C in a pathogen-free environment. The animals were fed conventional laboratory chow and had plenty of water available to them.

Histological examination and echocardiography

Rats were sedated with 3% isoflurane (v/v), and two dimensional-guided M-mode echocardiography was performed using a Technos MPX ultrasound system (Esaote, Genoa, Italy), as described in earlier investigations [1, 2]. The images were taken in M-mode and in 2D parasternal short-axis. Ejection fraction (EF), fractional shortening (FS), diastolic and systolic left ventricular posterior wall thickness (LVPW,d and LVPW,s) were all calculated. All animals were then sacrificed

as a result of this. For morphometric measurements, the hearts were stopped at end-diastole with a 0.1 mM KCl solution, fixed in 4 percent paraformaldehyde, and then calcinated.

Cell culture

Primary cultures of neonatal rat cardiomyocytes (NRCMs) were created using cells taken from the hearts of SD rats (1-2 days old) and identified using a specific test, as previously described[3]. Purified cardiomyocytes were seeded at a density of 1×10^6 cells per well into six-well plates, then maintained at 37°C with 5% CO_2 in DMEM supplemented with 10% FBS and 5-bromodeoxyuridine in DMEM supplemented with 10% FBS and 5-bromodeoxyuridine (0.1 mM). After 48 h, NRCMs were administered with isoprerenol or another stimulus before being incubated in serum-free medium for 24 h.

Western blot

SDS-polyacrylamide gel electrophoresis was used to separate equal amounts of total or mitochondrial protein, which was subsequently transferred to polyvinylidene fluoride membranes. The membranes were incubated with primary antibodies overnight at 4 °C before being exposed for 1 h to the appropriate secondary antibodies. The proteins were seen using enhanced chemiluminescence (Tanon, China).

Measurement of cell surface area

After being fixed with 4 % paraformaldehyde for 20 minutes at room temperature, cardiomyocytes were sown in 24-well plates and treated with 0.1 percent Triton X-100 for 5 minutes. After 1 h of incubation with 0.2 percent rhodamine phalloidin, the cells were washed with HBSS and stained with DAPI (Invitrogen, No. MAN0001777). The cell surface area was determined using installed image processing software from randomly picked fields utilizing a Screening System (Arrayscan VTi 600 Plus, Thermo Fisher Scientific) (30 for each group).

RNA extraction and quantitative real-time PCR (qRT-PCR)

The Trizol reagent was used to extract total RNA from rat cardiac tissues or primary cell cultures (Takara Biotechnology, Dalian, China), and 1 g of total RNA was reverse-transcribed with the First Strand cDNA Synthesis Kit (Takara Biotechnology, Dalian, China) (Thermo Fisher Scientific, USA). On an ABI7500 real-time PCR equipment, PCR was done using Master Mix (Takara, Dalian, China) (Applied Biosystems, Foster, CA, USA). The 2^{-Ct} technique was used to determine the relative expression level.

Subcellular fractionation and isolation of the MAM

After cervical dislocation was performed on Rats, the hearts were removed and placed in Starting Buffer. (SB: 225mM mannitol, 75mM sucrose, and 30mM Tris HCl, pH 7.4). Heart tissues were minced by scissors, followed by a homogenization using a tefon–glass grinder (25 strokes) with 5 ml of IB1 buffer (225 mM mannitol, 75 mM sucrose, 0.5% BSA, 30mM Tris Hcl and 0,5mM EGTA). The homogenates were centrifuged at 740 g/5 min/4 °C, and then, supernatants were collected and centrifuged at 8000 g/10 min/4 C. The pellets were gently resuspended in IB2 bufer

(225 mM mannitol, 75 mM sucrose, 0.5% BSA, and 30 mM Tris HCl, pH 7.4) and centrifuged at 10000 g/10 min/4 °C, while supernatants were preserved as cytosolic fractions. Then, pellets were resuspended again in SB to wash out the BSA and EGTA, followed by centrifugation at 10,000 g/10 min/4 °C. Crude mitochondria were resuspended in Mitochondria Resuspending Bufer (MRB: 250 mM Mannitol, 5 mM HEPES, 0.5 mM EGTA, pH 7.4). To purify the mitochondria and obtain mitochondria-associated membranes (MAMs), separation was done using a Percoll density gradient [225 mM Mannitol, 25 mM HEPES, 1 mM EGTA, and 30% Percoll (Sigma-Aldrich P1664)] in 14 ml thin-wall Beckman ultracentrifuge tubes, under 95,000 g/30 min/4 °C ultracentrifugation. Purifed mitochondria and MAMs, localized at the bottom of the tube as a dense band and in the middle as a round layer, respectively, were collected and washed by MRB at a centrifugation of 6500g /15 min/4 °C, and then, MAMs were re-centrifuged in polycarbonate tubes using the ultracentrifuge at 100,000 g/1 h/4 °C. A ring at the bottom of the tubes was collected, being the MAMs. For ER and cytosolic fractions, the cytosolic fractions were centrifuged at 20,000 g/30 min/4 °C to remove potential remaining mitochondria. Next, the supernatants were collected and re-centrifuged at 100,000 g/1 h/4 °C: pellets were saved as ER proteins and the supernatants as cytosolic ones.

Intramyocardial adenovirus injection

Animals were anesthetized and underwent a thoracotomy for intramyocardial injection of adenovirus vector harboring ATAD3A (Ad-ATAD3A) or green fluorescent protein (Ad-GFP, control; WZ Bioscience, Shandong, China). A total of 1×10^9 plaque-forming units per heart Ad-ATAD3A or Ad-GFP was delivered via direct myocardial injection using a 31-gauge needle attached to an

insulin syringe at 3 sites in the left ventricular free wall. After adenovirus administration, the chest was closed, and the animals recovered for 48 h. The targeted proteins were allowed to express for 48 h.

siRNA infection

siRNA were synthesized by GENECHEM (Shanghai, China). Cardiomyocytes were transfected with siRNA using TransIntro EL transfection reagent (TransGen Biotech, Beijing, China). Following the manufacturer's protocol for 72h before harvest. The sequence of siRNA-ATAD3A and siRNA-SIRT3 are shown in Supplementary Table S2 .

ATAD3A interactome

Cells were lysed in total lysis buffer (10 mM HEPES, pH 7.4, 150 mM NaCl, 1 mM EGTA, 1% NP-40, and protease inhibitor cocktail). Three milligram of the lysates were immunoprecipitated using anti-ATAD3A antibody overnight followed by 4 h incubation with protein A/G beads. Bound proteins were eluted by 0.2 M glycine (pH 2.5). The proteins from each sample were then precipitated using 30% trichloroacetic acid (v/v), and the protein pellet was washed using ice-cold acetone. The pellet was air-dried, and the proteins were then trypsinized and analyzed by tandem mass spectrometry. Digested peptides were reconstituted with 0.1% formic acid and analyzed by LC-MS/MS. Isotopic C13 incorporated ions were automatically included. DTASelect was applied to generate search results of peptide-to-spectra matches (PSMs) with a max false discovery rate (FDR) of 5%, yielding a protein FDR of less than 1% with at least two peptides per protein being

assigned. Enriched mitochondrial candidates of ATAD3A-interacting proteins were analyzed using Ingenuity IPA software.

Surface plasmon resonance measurement (SPR)

The SPR experiments were performed on a Biacore 8K instrument (GE Healthcare, CA). Recombinant proteins ATAD3A served as the ligand and were, respectively, immobilized on a gold nanoparticle sensor chip via capture-coupling. Subsequently, the recombinant protein SIRT3 at different concentrations were sequentially injected into the chamber in running buffer (filtered PBS) with a constant flow rate of 20 μL/min, and were passed over the sensor (about 5 min) for the association of two protein. Following each recombinant protein injection (all concentrations were performed in triplicate), the chip was completely dissociated with the complex and regenerated by injecting hydrochloric acid. As recommended by the manufacturer, the results were analyzed by Biacore 8 K evaluation software. The kinetic parameters, including the association constant (ka), dissociation constant (kd) and affinity (KD, KD = kd/ka), were calculated by a simple 1:1 dilution corrected model, which adjusted to the wavelength shifts consisting with the varied concentration of protein.

Bimolecular fluorescence complementation assay (BiFC)

In brief, the coding fragment of SIRT3 was inserted into the piBC-VN155 vector, and the coding fragments of ATAD3A were inserted into the piBC-VC155 vector, respectively. Subsequently, constructs were co-transformed into NRCMs and incubated for 24 h. Images were acquired with a

confocal microscope at the EYFP channel. Bar: 10 μm. YFP fluorescence signals were captured using a fluorescence microscope (Olympus, Tokyo, Japan).

FRET

NRCMs were seeded and transfected with either 0.5 μg of EGFP-ATAD3A and 0.5 μg of mCherry-SIRT3. The acceptor was bleached using 20 to 50% power of the 750-nm laser and bleached using two-photon excitation to achieve photobleaching. The width of the bleached region was 2 μm. FRET acceptor photobleaching mode of Olympus Imaging software was used, with the following parameters: acquisition of 5 pre-bleach images, one bleach scan and 50 post-bleach scans.

FRAP

FRAP was performed using the FRAP module on Olympus Confocal microscope Software. For FRAP, NRCMs were incubated with 100 nM TMRE for 30 min in the dark. Bleaching was performed during fly forward using ROI scan features. In the FRAP experiments, spherical areas of approximately 2 μm diameter were photobleached for 500 msec, and subsequently, images of the area were collected approximately every 4 frames/s. Quantification of fluorescence intensity for FRAP was done using ImageJ.

Protein-protein docking

Single chain structure of SIRT3 and ATAD3A were obtained with PyMol software. Amino acid residues sequence of SIRT3 derived from 3GLS.pdb, and the 3D structural model of the ATAD3A was predicted by SWISSMODEL software (http://swissmodel.expasy.org) and graphically

rendered using the PyMol. SIRT3-ATAD3A docking was examined using the rigid-body docking program ZDOCK 3.0.2 on the ZDOCK server (http://zdock.umassmed.edu). Interface residues of the best SIRT3-ATAD3A complex were analysed by PDBePISA website (https://www.ebi.ac.uk/msd-srv/prot_int/ pistart.html).

Mitochondrial fission Analysis

NRCMs were washed in PBS and then incubated for 30 minutes at 37 °C with 300 nmol/L Mito Tracker red (Molecular Probes). Confocal microscopy was used to examine the structure of mitochondria. The percentage of cells undergoing mitochondrial fission was calculated from 90 cells in three separate studies. The detection and statistics of mitochondrial fission were carried out.

GST pulldown assay.

GST-ATAD3A fusion protein and truncated GST-ATAD3A protein were added to neonatal rat cardiomyocyte lysate and mixed at 4°C overnight. GST protein was used as a negative control. After incubating the mixture with GST-beads (Beyotime Institute of Biotechnology, Jiangsu, China) for another 2 h at 4°C, beads were harvested by centrifugation and washed four times with 0.3% NP-40 buffer. Samples were boiled in SDS loading buffer and subjected to Western blotting analysis. The beads were washed 3 times with washing buffer and then denatured by the addition of 2× loading buffer, boiled 5 min and analysed by Western blot.

Measurement of mitochondrial oxygen consumption rate (OCR)

OCR was measured using an XF96 Extracellular Flux Analyzer (Seahorse Bioscience, North Billerica, MA, USA). H9c2 cells were seeded at a density of 5×10^4 cells/well in Seahorse XF96 microplates (Seahorse Bioscience) for 24 h in a 37 °C incubator with 5% CO_2 (DMEM). The cells' regular medium was then changed with XF assay medium, which had 1 nM pyruvate, 10 mM glucose, and 2 mM glutamine. The following substances were used to fill a sensor cartridge: oligomycin (2 M), carbonylcyanide 4-(trifluoromethoxy) phenylhydrazone (FCCP, 1 M), and rotenone/antimycin A (1 M). The cell plates were incubated in a 37 °C/non-CO_2 incubator for 60 minutes prior to the start of the OCR test after the sensor cartridge was calibrated in the analyzer. The OCR was normalized using the cell count in each well. The data were analyzed with Wave software, and respiratory parameters were quantified. At least three times, the experiment was carried out.

Determination of mitochondrial membrane potential (ΔΨm) and ATP production

The methyl ester of tetramethylrodhamine was used to determine the mitochondrial membrane potential (TMRM, Invitrogen). Cardiomyocytes were collected, washed, and resuspended in PBS, in a nutshell. After that, cells were loaded with 200 nM TMRM in PBS for 20 minutes at 37 °C. A FACScan flow cytometer was used to assess the cells after they had been washed (Beckman Coulter). A total of 10,000 occurrences were captured in each analysis. A luciferase-based assay kit was used to assess the amounts of ATP in NRCMs (Beyotime, Shanghai, China). NRCMs were lysed to extract ATP from within the cells in a nutshell. Based on the ATP and luciferase responses, the ATP

level was evaluated using a mix of ATP detection solution and luciferase solution. The concentration was adjusted to match the cellular protein concentration. The final outcomes were revealed. The final percentage of the control group was presented.

Intracellular calcium measurements

Cells were seeded on 35 mm imaging dishes (Ibidi, Martinsried, Germany), transfected with a cameleon sensor targeted to the mitochondria (pcDNA-4mtD3cpv), and, in case of cardiomyocytes, co-transfected with siRNAs. Cells were incubated in phenol red-free medium supplemented with 2 mmol/l L-glutamine and antibiotics in the absence or presence of serum for 24 h before imaging. Images were acquired using FV3000 laser scanning confocal microscope (Olympus Life Science). Fluorescence emission was monitored at 482/35 nm (CFP) and 540/30 nm (YFP) following excitation at 445 nm, and the ratio (R) of YFP and CFP fluorescence intensity was quantified after background-subtraction and threshold setting. The resulting images were displayed in pseudocolor. IP3R-mediated calcium release from ER stores was triggered by the addition of 100 lmol/l histamine or 100 lmol/l ATP. Caffeine (to stimulate calcium release via ryanodine receptors) was added at a final concentration of 10 mmol/l immediately before recording. In this set of experiments, mitochondrial calcium levels were calculated as relative YFP/CFP ratio compared to baseline ratio at the start of the measurement (% R/R0). For ER calcium measurements, we used a cameleon sensor targeted to the ER (pcDNA-4D1ER). Cells were incubated in HBSS (Thermo Fisher Scientific) supplemented with 2 mmol/l L glutamine and antibiotics in the absence of serum before imaging and detection of fluorescence emission as described above. ER calcium levels were calculated as relative YFP/CFP ratio compared to baseline ratio at the start of the measurement (%

R/R0). Cytosolic calcium measurements were performed using Fluo4- AM. Cells were loaded with 2 mol/l Fluo4-AM (Thermo Fisher Scientific) in solution containing 0.02% Pluronic F27 (Thermo Fisher Scientific) for 30 min at 37°C. Fluo4 (green) luorescence was recorded (at 1s intervals) at 37 °C. Fluo 4 was excited at 488 nm and emission visualized at 525/40 nm. Cytosolic calcium levels were calculated as relative Fluo4-AM fluorescence compared to baseline fluorescence at the start of the measurement (% F/F0).

Transmission electron microscopy (TEM)

To determine the structure of mitochondria morphologically, ultrastructure analysis was conducted out as previously reported. The heart tissue was initially sliced into 1mm^3 patches and treated with ice-cold glutaraldehyde at a concentration of 2.5 percent (solarbio, Beijing, China). After being washed in PBS, all samples were implanted in epoxy resin after being post-fixed with percent OsO4 (Electron Microscopy Sciences, Hatfield, PA, USA). Uranyl acetate (E. Merck, Darmstadt, Germany) and lead citrate were used to stain ultrathin slices (Sigma-Aldrich, St. Louis, MO, USA). For observation, a transmission electron microscope (JEM-100CXII, Japan) was employed. The entire procedure was carried out with the observer obscured from view. The statistical analysis was carried out using Graphpad Prism 9.0.

Materials

Primary antibodies against SIRT3 (Cat# 5490), Drp1 (Cat# 14647), Acetylated-Lysine (Cat# 9441) were obtained from Cell Signaling Technology. Antibodies against PERK (sc-377400), CHOP (sc-7351), IP3R1 (sc-271197), were purchased from Santa Cruz Biotechnology (Dallas, TX, USA).

Beta-MHC antibody was obtained from Sigma-Aldrich. Actin (Cat# 60004-1-AP), P-PERK (Cat# 29546-1-AP), GRP75 (Cat# 14887-1-AP), VDAC1 (Cat# 55259-1-AP), FACL4 (Cat# 22401-1-AP) bodies were purchased from Proteintech Group (Manchester, UK). MCU and MICU1 antibody were purchased from Affinity Biosciences (Cincinnati, OH, USA).

Statistical analysis

The data are expressed as the mean ± SEM of multiple independent replicates. The statistical analyses included two-tailed, unpaired Student's t tests for experiments with two groups or one-way ANOVA with Bonferroni posttests for multiple groups (GraphPad Prism 9.0 software, San Diego, CA, USA). Statistical significance was defined as $P < 0.05$.

Supplementary Table S1. Primer sequences for qRT-PCR

Gene	Primer sequences
beta-Actin	Forward: 5'-GCAGATGTGGATCAGCAAGC-3'

	Reverse: 5'-GCAGCTCAGTAACAGTCCGC-3'	
SIRT3	Forward: 5'-CCTGTCTGTACTGGCGTTGT-3'	
	Reverse: 5'- AGTCGGGGCACTGATTTCTG-3'	
beta-MHC	Forward: 5'-TTACTTGCTACCCTCAGGTGG-3'	
	Reverse: 5'-CTCCTTCTCAGACTTCCGCA-3'	
ANF	Forward: 5'-CTGGGACCCCTCCGATAGAT-3'	
	Reverse: 5'-CACTCTGGGCTCCAATCCTG-3'	
BNP	Forward: 5'-GGCTGTAACGCACTGAAGTT-3'	
	Reverse: 5'-CACTTCAAAGGTGGTCCCAG-3'	
ATAD3A	Forward: 5'-CGATGGATTCCCGTTCGAGT-3'	
	Reverse: 5'-CGATCTCGTTGGATCCCTGG-3'	

Supplementary Table S2. The sequences used for RNA interference analyses

Gene Name	Sequence
sgRNA-ATAD3A#1	5'-CACCGAATGAGATGCTGCGAGTGG-3'
sgRNA-ATAD3A#2	5'-CACCGCCCCAAGGGTGAAGGCGCG-3'
sgRNA-ATAD3A#3	5'-CGGAATTCGTCGTGGCTCTTCGGCAT-3'
siRNA-ATAD3A#1	5'-GACACUAUUUGCUAAGAAACU -3'

siRNA-ATAD3A#2	5'- CCAACGUGAAGAAGGAAAUTT-3'
siRNA-ATAD3A#3	5'- CCUCAGGGCUACUCUGAAUTT-3'
siRNA-SIRT3#1	5'-GCGUUGUUGAAACCUGACAUTT-3'
siRNA-SIRT3#2	5'-CAGCAAGGUUCUUACUACATT-3'

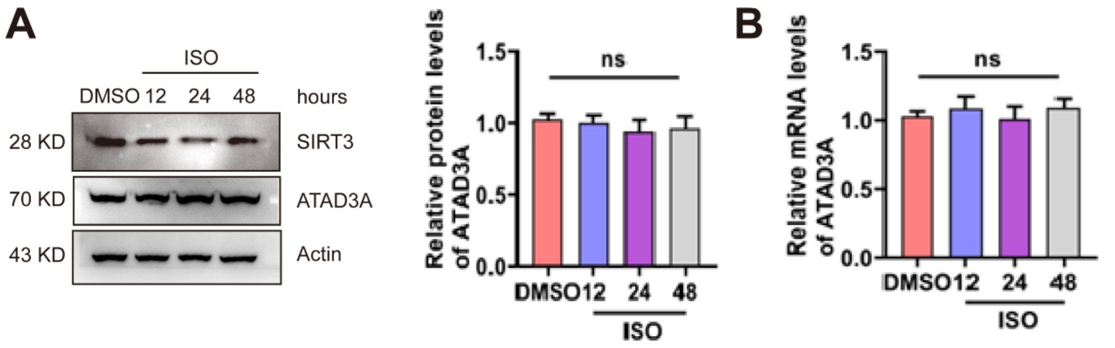

Figure S1 The protein and mRNA levels of ATAD3A after stimulation of NRCMs with ISO. **(**A) NRCMs were exposed to ISO (10 μM) at different time point (0, 12, 24 and 48 h). The protein level of SIRT3 and ATAD3A was detected by Western blot. The protein levels of ATAD3A in NRCMs were measured by Western blot analysis. (B) The mRNA levels of ATAD3A in NRCMs were measured by qRT-PCR. The data are presented as the mean ± SEM.

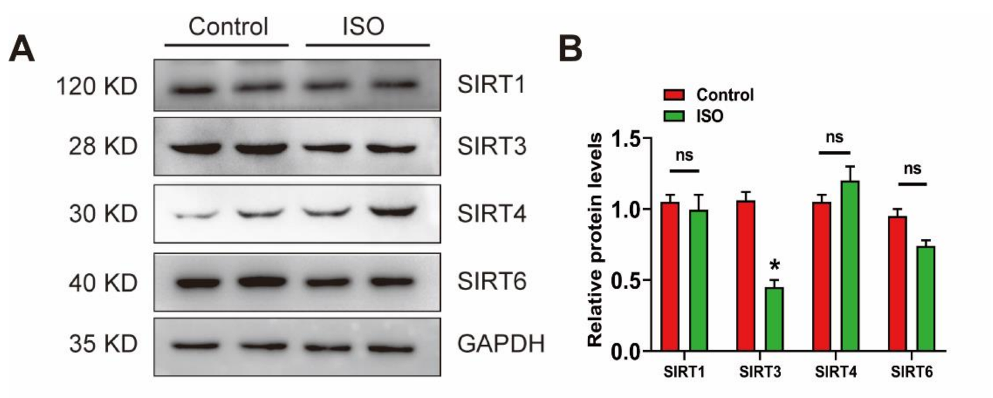

Figure S2 Changes in the expression of SIRT family proteins under ISO stimulation. NRCMs were treated with with Ad-GFP or Ad-ATAD3A for 48 h, followed by incubation with ISO (10 μM, 24 h). (A) The protein expression of SIRT family proteins were detected by western blot analysis. (B) The data are presented as the mean ± SEM. **P*< 0.01, *vs*. the Control group; n=3.

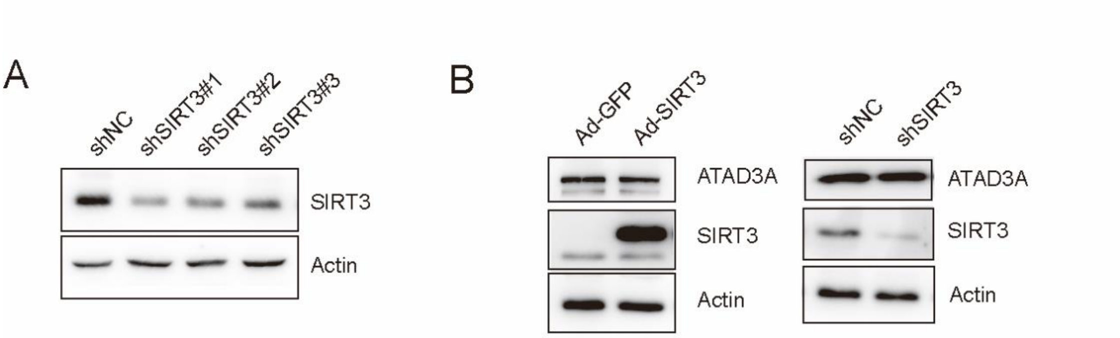

Figure S3 SIRT3 upregulation has no effect on the expression level of ATAD3A in NRCMs. (A) The protein expression of SIRT3 was detected in NRCMs by Western blot. Representative bands were shown. (B) The protein expression of SIRT3 and ATAD3A was detected in NRCMs by Western blot. Representative bands were shown.

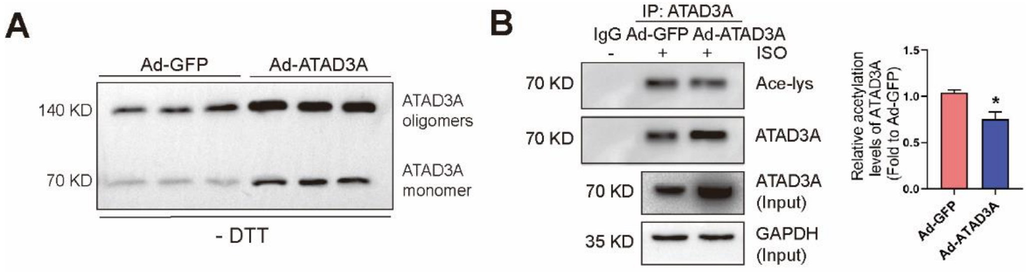

Figure S4 Overexpression of ATAD3A affect its acetylation and oligomerization. (A) ATAD3A oligomers were analyzed in NRCMs transfected with Ad-GFP or Ad-ATAD3A, followed by incubation with ISO (10 μM, 24 h). (B) Analysis of acetylation of ATAD3A in NRCMs transfected with Ad-GFP and Ad-ATAD3A, followed by incubation with ISO (10 μM, 24 h). The data are presented as the mean ± SEM. $^{*}P < 0.01$, n = 3.

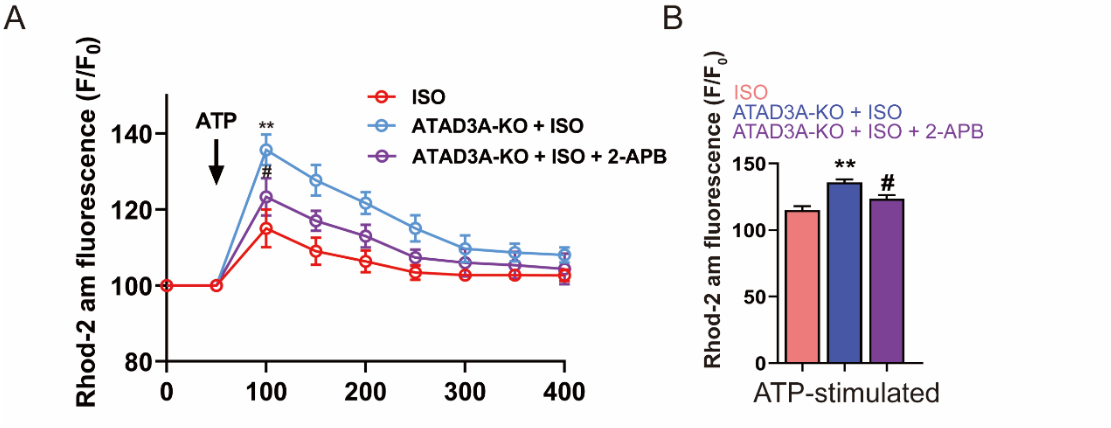

Figure S5 2-APB inhibits mitochondrial calcium influx overload caused by ATAD3A dysregulation. Mitochondrial Ca^{2+} flux was measured using Rhod2-AM. $[Ca^{2+}]_{mito}$ after stimulation with 500 nmol/L ATP is shown.

1. Li J, Huang J, Lu J, et al. Sirtuin 1 represses PKC-ζ activity through regulating interplay of acetylation and phosphorylation in cardiac hypertrophy. Brit J Pharmacol. 2019;176:416-435.

2. Wang P, Wang L, Lu J, et al. SESN2 protects against doxorubicin-induced cardiomyopathy via rescuing mitophagy and improving mitochondrial function. J Mol Cell Cardiol. 2019;133:125-137.

3. Fu J, Gao J, Pi R, Liu P. An Optimized Protocol for Culture of Cardiomyocyte from Neonatal Rat. Cytotechnology. 2005;49:109-116.

RAW Western Blots

FIGURE1

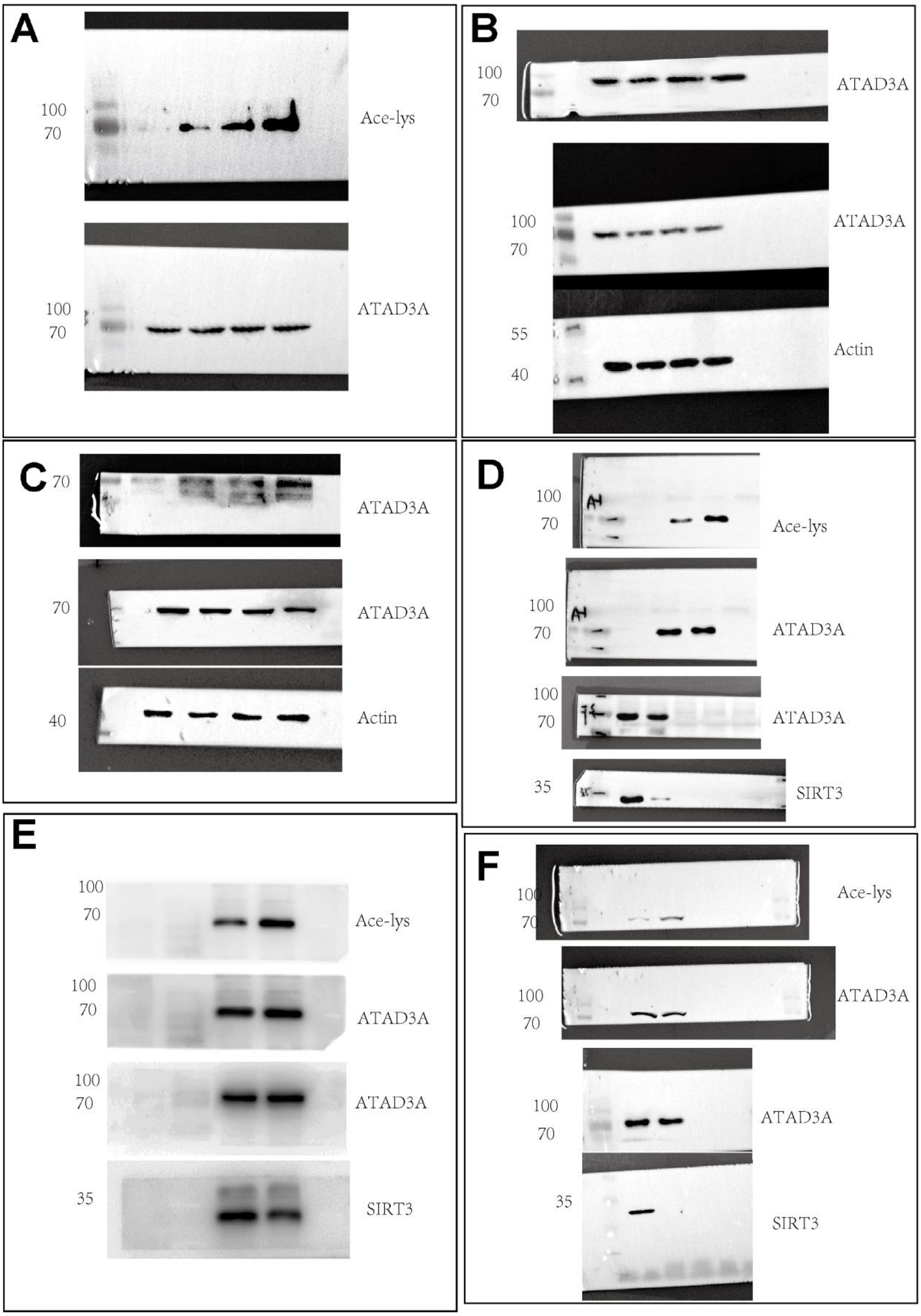
A
100
70
Ace-lys
100
70
ATAD3A
B
100
70
ATAD3A
100
70
ATAD3A
55
40
Actin
C
70
ATAD3A
70
ATAD3A
40
Actin
D
100
70
Ace-lys
100
70
ATAD3A
100
70
ATAD3A
35
SIRT3
E
100
70
Ace-lys
100
70
ATAD3A
100
70
ATAD3A
35
SIRT3
F
100
70
Ace-lys
100
70
ATAD3A
100
70
ATAD3A
35
SIRT3

FIGURE2

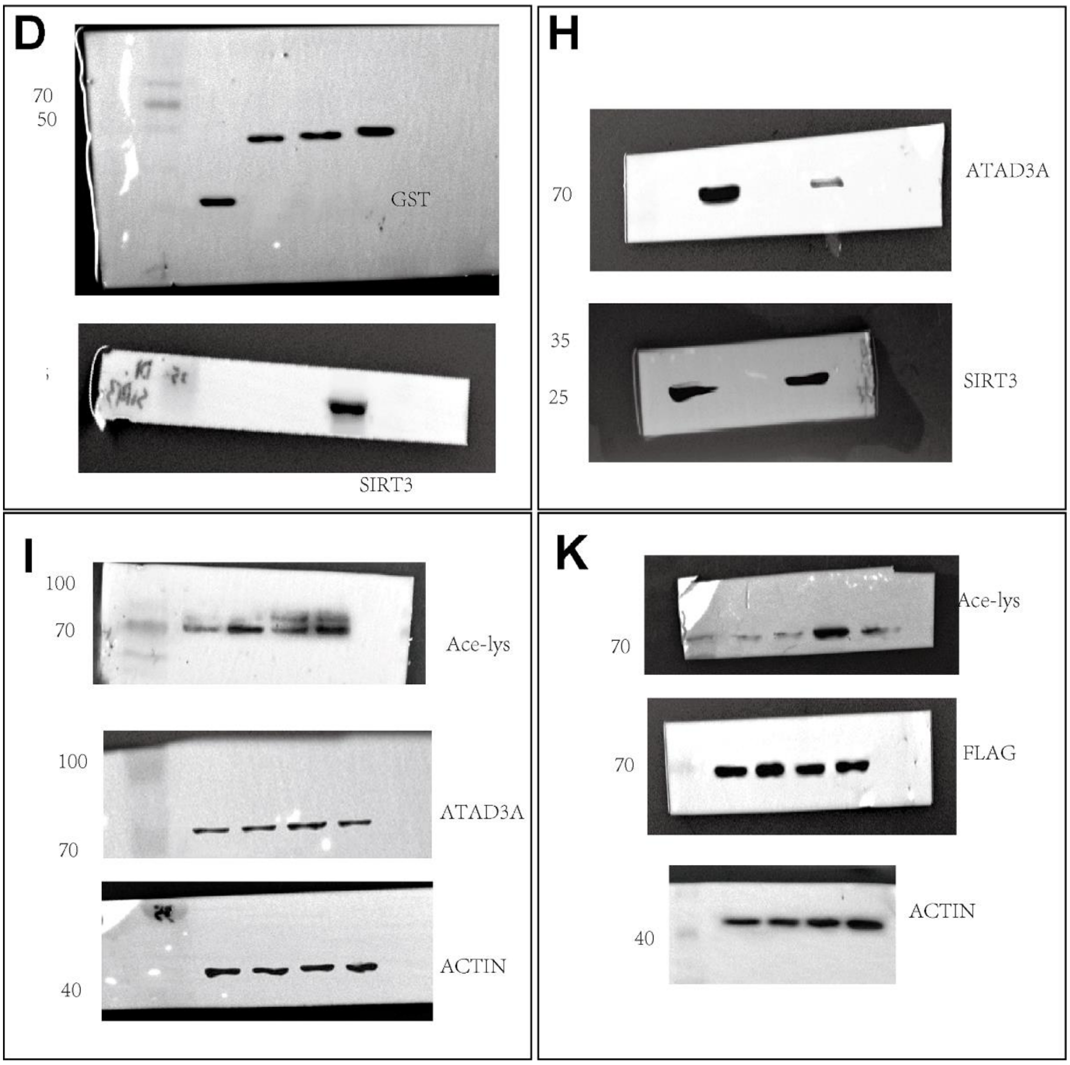
D
70
50
GST
SIRT3
H
ATAD3A
70
35
25
SIRT3
I
100
70
Ace-lys
100
ATAD3A
70
ACTIN
40
K
Ace-lys
70
70
FLAG
ACTIN
40

FIGURE3

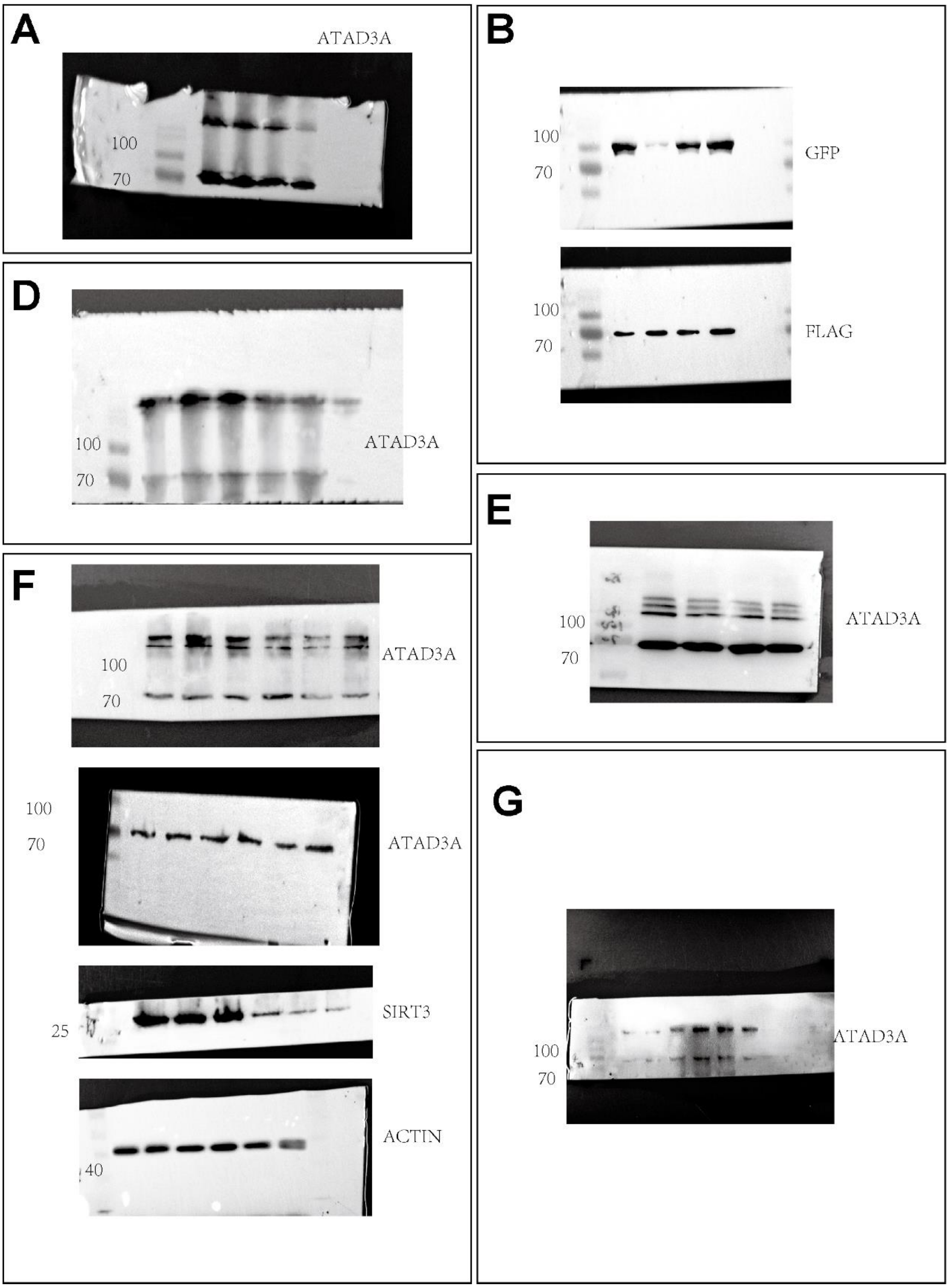
A
ATAD3A
100
70
B
100
70
GFP
100
70
FLAG
D
100
70
ATAD3A
E
100
70
ATAD3A
F
ATAD3A
100
70
100
70
ATAD3A
SIRT3
25
ACTIN
40
G
ATAD3A
100
70

FIGURE4

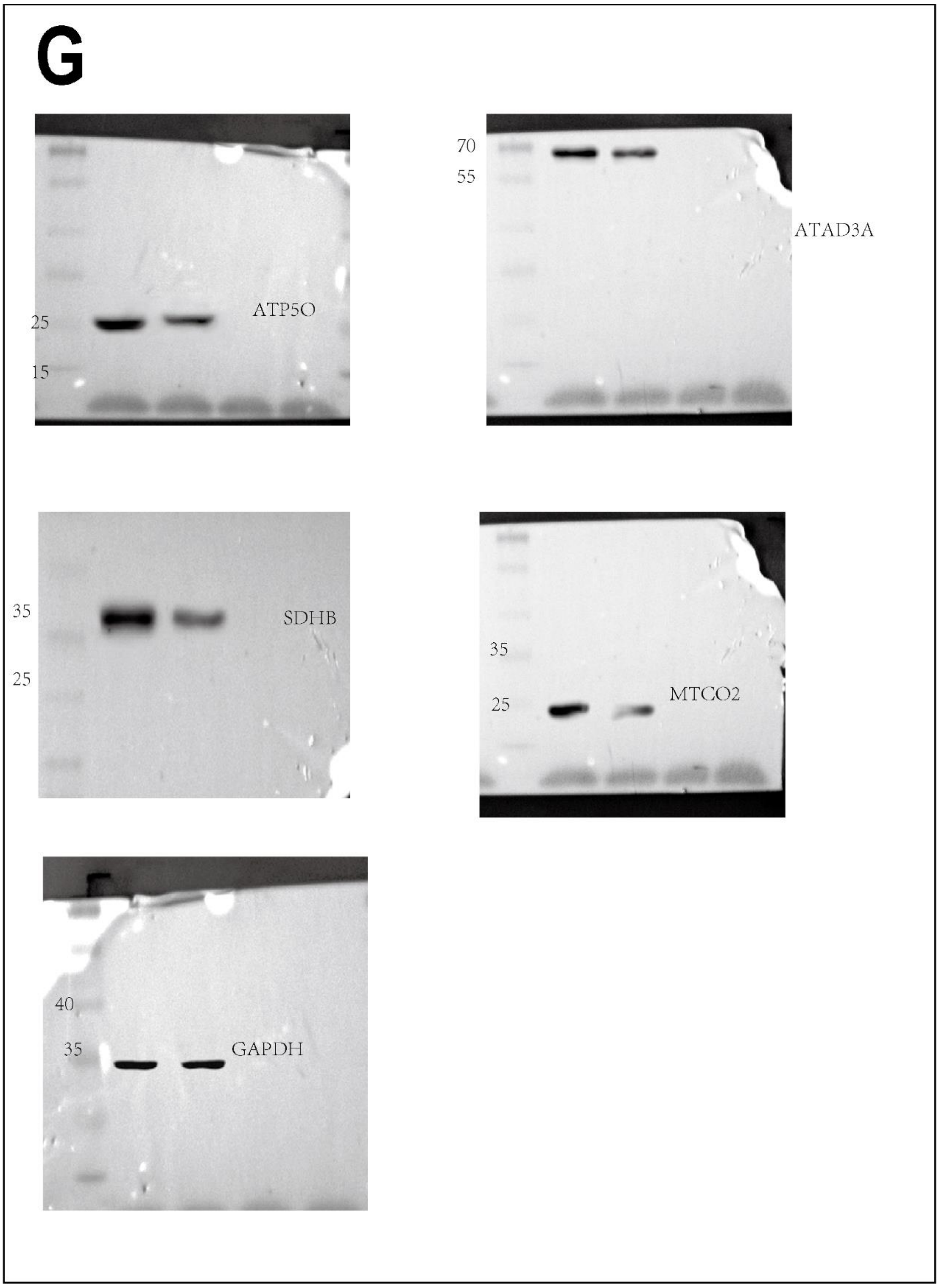

FIGURE5

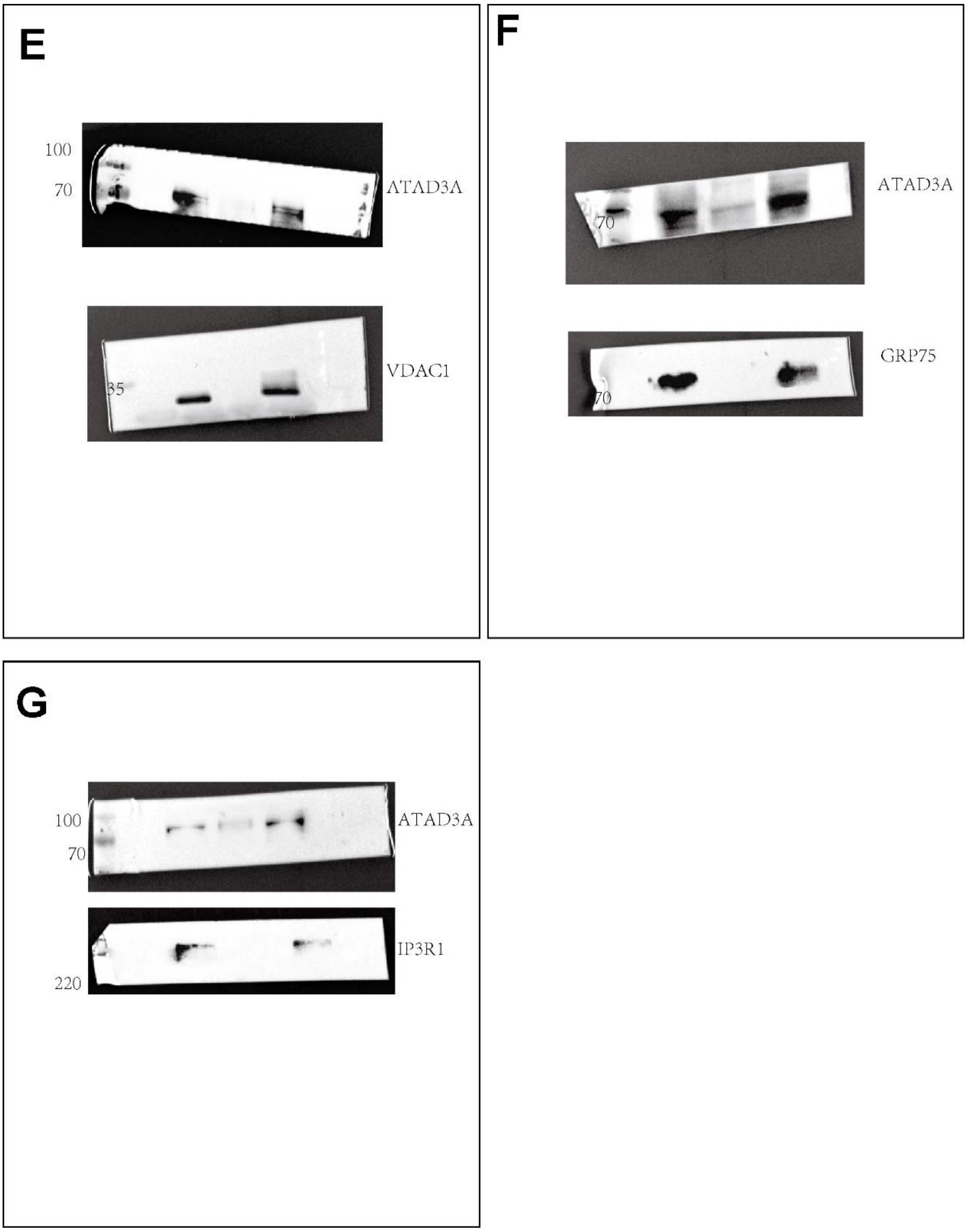
E
100
70
ATAD3A
VDAC1
35
F
ATAD3A
70
GRP75
70
G
100
70
ATAD3A
IP3R1
220

FIGURE6

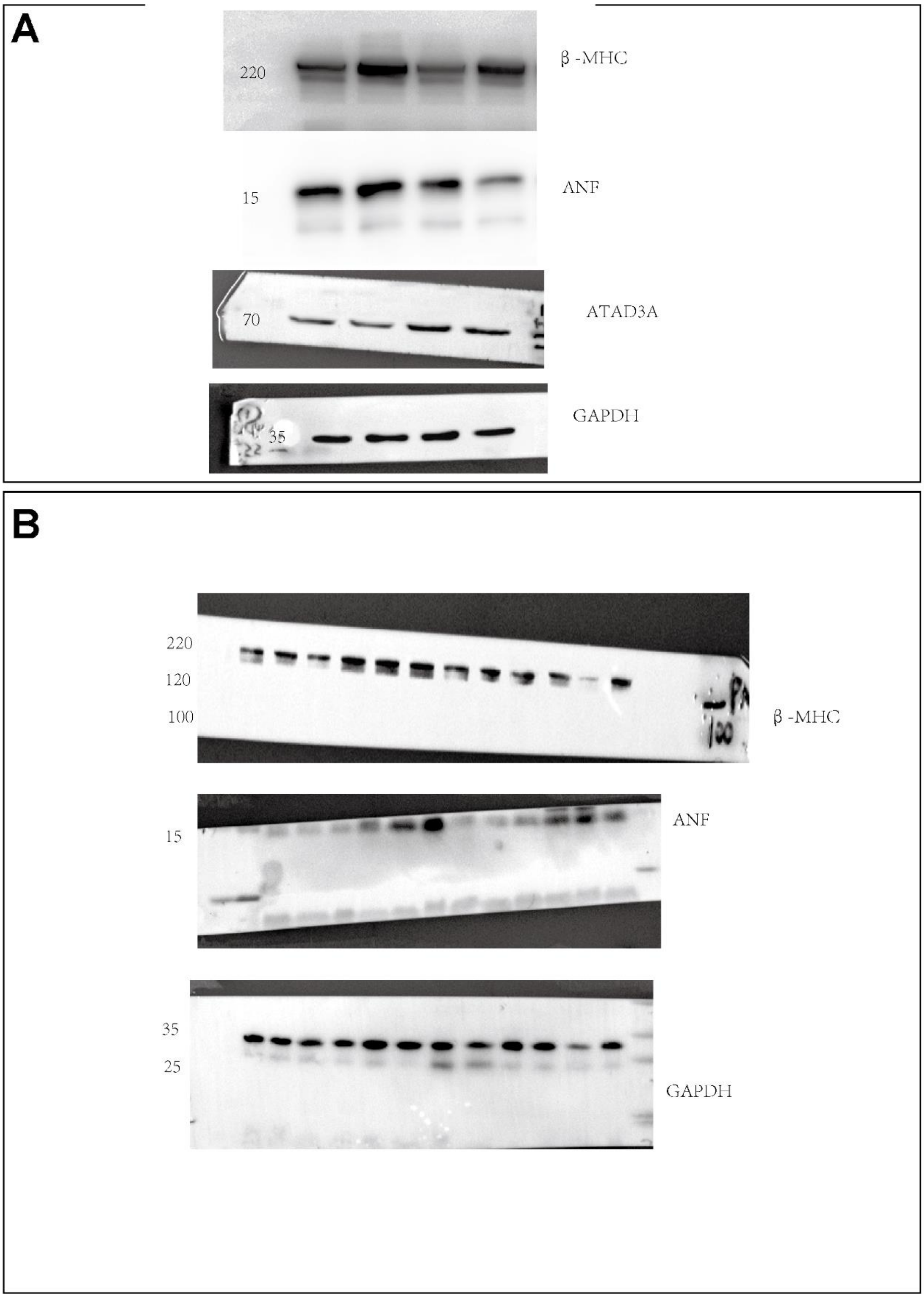
A
220
β -MHC
15
ANF
70
ATAD3A
35
GAPDH
B
220
120
100
β -MHC
15
ANF
35
25
GAPDH

FIGURE7

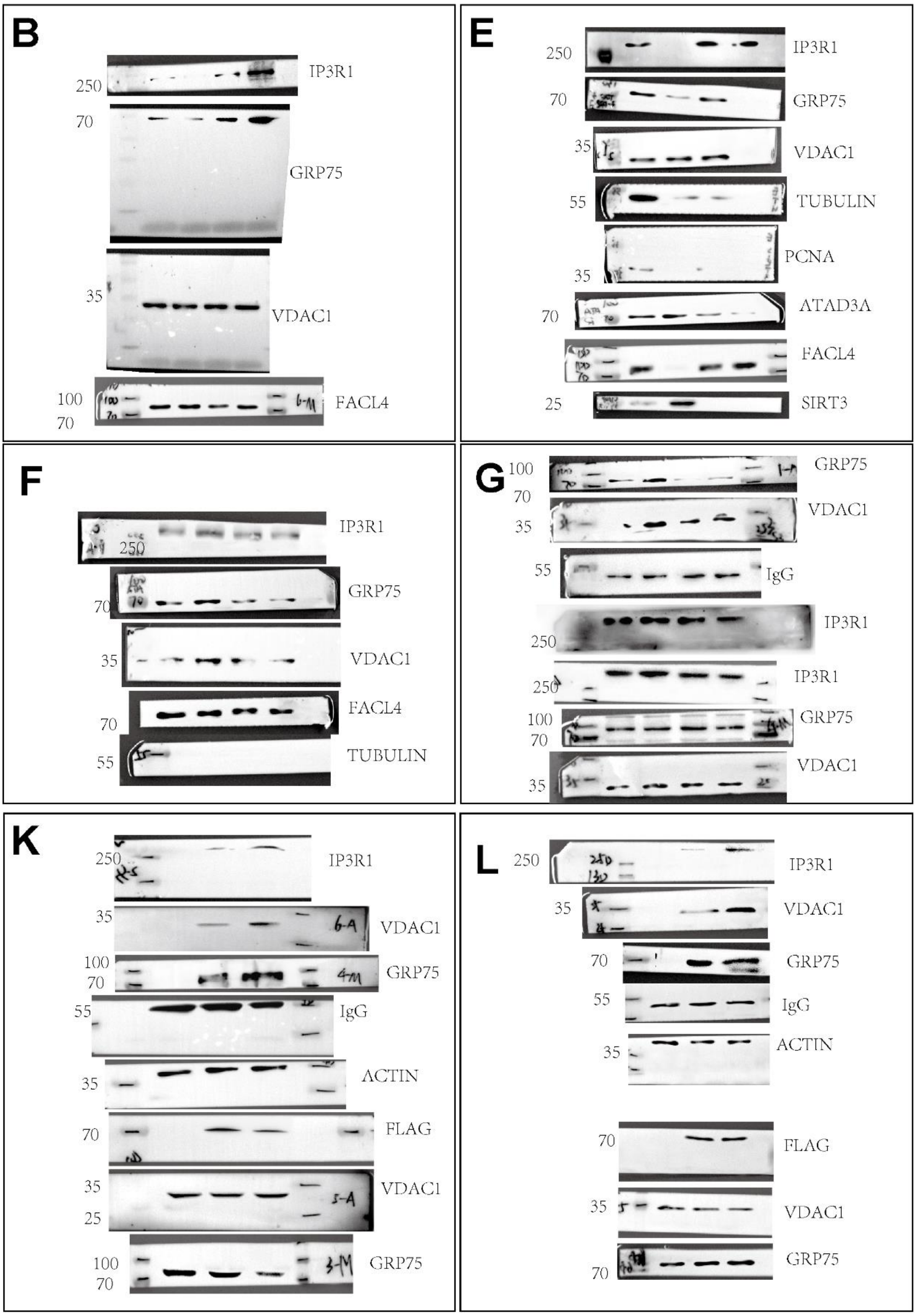
B
IP3R1
250
70
GRP75
35
VDAC1
100
70
FACL4
E
250
IP3R1
70
GRP75
35
VDAC1
55
TUBULIN
PCNA
35
70
ATAD3A
FACL4
25
SIRT3
F
IP3R1
250
GRP75
70
35
VDAC1
70
FACL4
55
TUBULIN
G
100
70
GRP75
35
VDAC1
55
IgG
IP3R1
250
250
IP3R1
100
70
GRP75
VDAC1
35
K
250
IP3R1
35
VDAC1
100
70
GRP75
55
IgG
35
ACTIN
70
FLAG
35
25
VDAC1
100
70
GRP75
L
250
IP3R1
35
VDAC1
70
GRP75
55
IgG
35
ACTIN
70
FLAG
35
VDAC1
70
GRP75

FIGURE8

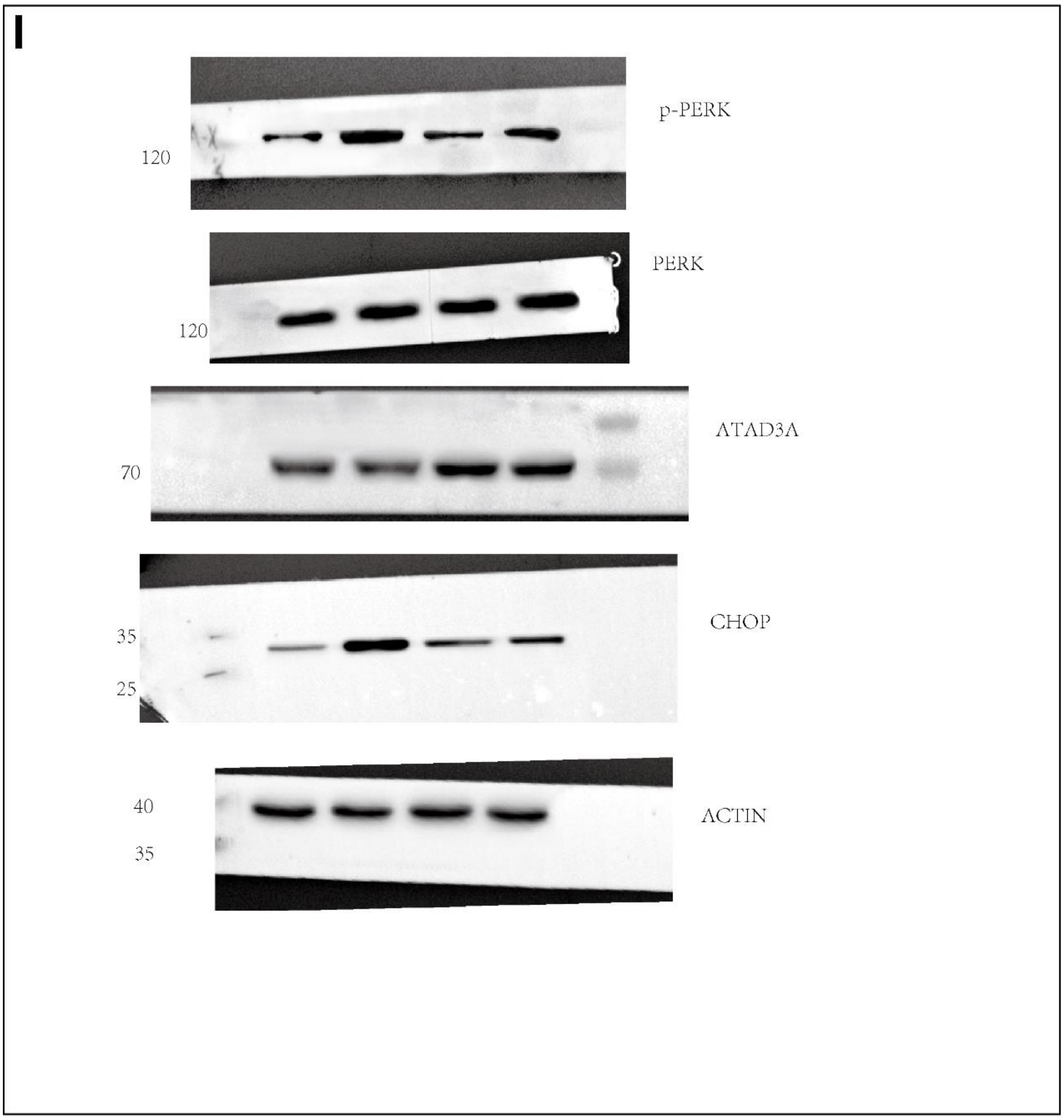
I
p-PERK
120
PERK
120
ATAD3A
70
CHOP
35
25
ACTIN
40
35

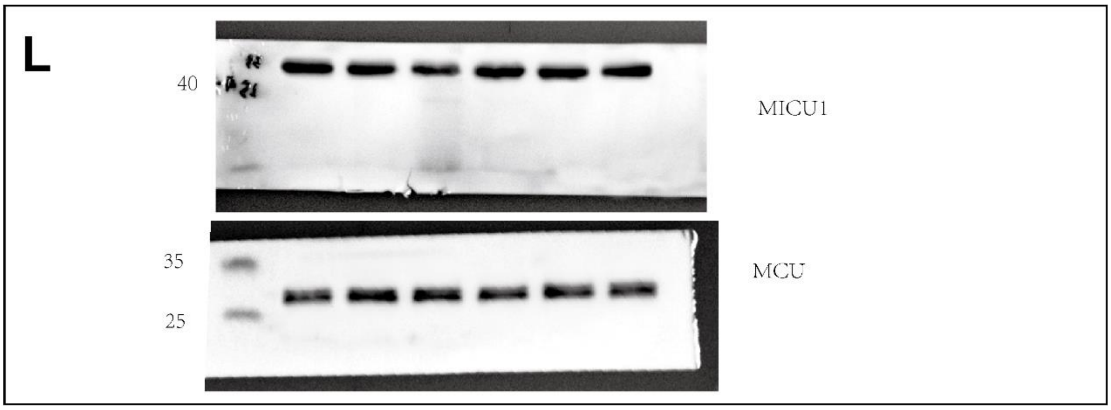
L
40
MICU1
35
MCU
25
